# Mask, the *Drosophila* ankyrin repeat and KH domain-containing protein, affects microtubule stability

**DOI:** 10.1242/jcs.258512

**Published:** 2021-10-22

**Authors:** Daniel Martinez, Mingwei Zhu, Jessie J. Guidry, Niles Majeste, Hui Mao, Sarah T. Yanofsky, Xiaolin Tian, Chunlai Wu

**Affiliations:** 1 Neuroscience Center of Excellence, Department of Cell Biology and Anatomy, Louisiana State University Health Sciences Center, New Orleans, LA 70112, USA; 2 Proteomics Core Facility, and the Department of Biochemistry and Molecular Biology, Louisiana State University Health Sciences Center, New Orleans, LA 70112, USA

**Keywords:** *Drosophila*, Mask, Microtubule Stability

## Abstract

Proper regulation of microtubule (MT) stability and dynamics is vital for essential cellular processes, including axonal transportation and synaptic growth and remodeling in neurons. In the present study, we demonstrate that the *Drosophila* ankyrin repeat and KH domain-containing protein Mask negatively affects MT stability in both larval muscles and motor neurons. In larval muscles, loss-of-function of *mask* increases MT polymer length, and in motor neurons, loss of *mask* function results in overexpansion of the presynaptic terminal at the larval neuromuscular junctions (NMJs). *mask* genetically interacts with *stathmin* (*stai*), a neuronal modulator of MT stability, in the regulation of axon transportation and synaptic terminal stability. Our structure–function analysis of Mask revealed that its ankyrin repeats domain-containing N-terminal portion is sufficient to mediate Mask's impact on MT stability. Furthermore, we discovered that Mask negatively regulates the abundance of the MT-associated protein Jupiter in motor neuron axons, and that neuronal knocking down of *Jupiter* partially suppresses *mask* loss-of-function phenotypes at the larval NMJs. Taken together, our studies demonstrate that Mask is a novel regulator for MT stability, and such a role of Mask requires normal function of Jupiter.

## Abstract (precis)

**Summary:** Mask is a novel regulator of MT stability in *Drosophila*. Mask shows prominent interplay with two important modulators of MT, Tau and Stathmin (Stai), whose mutations are related to human diseases.

## INTRODUCTION

Terminally differentiated post-mitotic cells, such as neurons and muscles, use their microtubule (MT) network not for cell division but rather as architectural components essential for their shape and unique cellular functions. In neurons, in addition to supporting the structural integrity of the axons and the dynamic morphological changes of the dendrites, MTs also act as directional railways for transporting materials and organelles between the cell bodies and the synapses (Kapitein and Hoogenraad, 2015). MTs can undergo cycles of dynamic assembly and disassembly (labile state) or stay relatively stable depending on the cellular contexts (van der Vaart et al., 2009). For example, MTs in post-mitotic neurons are generally more stable than MTs in dividing cells. However, within a developing neuron, MTs at the axon growth cone are much more labile than MTs near the soma (Hur et al., 2012), and additionally, in individual axons, the MT network consists of domains that differ in their stability (Baas et al., 2016). Both the stable and labile pools of MTs play essential roles for normal neuronal functions, and the spacing among MTs and distance between MT ends have both been shown to be critical for normal axonal transport (Hahn et al., 2019; Yogev et al., 2016). Therefore, striking a balance in MT stability is of vital importance to maintaining MT-mediated cellular functions.

Many proteins and pathways have been identified as potential regulators of MT stability (Bowne-Anderson et al., 2015; van der Vaart et al., 2009). Among the major proteins controlling MT stability, Stathmin and Tau (MAPT) are both MT-binding proteins that regulate multiple aspects of MT stability, including growth and shrinkage as well as the transition between catastrophe and rescue. Both Stathmin and Tau are also associated with diverse models of neurodegeneration, axon transport defects, and cancer (Rossi et al., 2018; Strang et al., 2019; Tararuk et al., 2006; Wen et al., 2010). Whereas *in vitro* studies of Stathmin-related proteins in mammals suggest that Stathmin promotes destabilization of MTs, studies of *stathmin* (*stai*) in fly neuromuscular junction (NMJs) showed that it is required for MT stabilization, axon transport, and NMJ stability (Duncan et al., 2013; Graf et al., 2011). The results of the *in vivo* studies are consistent with the fly data in that *stathmin* knockout mice exhibit age-dependent axonopathy in both the central and peripheral nervous systems, and they exhibit defective motor axon outgrowth and regeneration (Klim et al., 2019; Liedtke et al., 2002). Tau, on the other hand, plays a multifaceted role in cell survival signaling. Loss of Tau function or high levels of hyperphosphorylated Tau disrupts MT stability, leading to axonal transportation defects in motor neurons and MT breakdown in larval muscles (Xiong et al., 2013). Additionally, hyperphosphorylated Tau aggregates form inclusion bodies associated with a variety of disorders collectively referred to as tauopathies, including Alzheimer's disease (Braak and Braak, 1991). In animal models, such as rodents and fruit flies, overexpression of human Tau in the neuronal tissues leads to progressive neurodegeneration (Wittmann et al., 2001).

Mask is a 4001-amino-acid protein with several functional domains. It consists of two ankyrin repeats, a nuclear export signal (NES), a nuclear localization signal (NLS) and one C-terminal KH domain. The two ankyrin repeats domains contain 15 and 10 tandem ankyrin repeats and probably facilitate the ability of Mask to associate with other proteins according to the well-documented involvement of the ankyrin domains in mediating protein–protein interactions in eukaryotic cells (Mosavi et al., 2004). The NES and NLS motifs may be required for shuttling Mask protein in and out of the nucleus, which is essential for its interaction with the Hippo pathway effector Yorkie (also known as YAP in mammals) in mitotic cells (Sansores-Garcia et al., 2013; Sidor et al., 2019; Sidor et al., 2013). The KH domain is an evolutionarily conserved motif that is about 70 amino acids long, and it was first identified in the human heterogeneous nuclear ribonucleoprotein K (Musco et al., 1997). KH domains bind RNA or single-stranded DNA (ssDNA) and are found in proteins involved in transcription, translation and mRNA stability regulation (Garcia-Mayoral et al., 2007; Grishin, 2001). Mask has been linked to several signaling pathways and different cellular processes in mitotic cells: it regulates the growth and morphology of the fly eye (DeAngelis et al., 2020; Smith et al., 2002); and it is a component of the centrosome and nuclear matrix (Kallappagoudar et al., 2010; Müller et al., 2010) and a co-transcription factor of the Hippo pathway (Sansores-Garcia et al., 2013; Sidor et al., 2013). The human homolog of Mask, ANKHD1, is expressed at relatively high levels in acute leukemia cells (Traina et al., 2006), multiple myeloma cells (Dhyani et al., 2012) and prostate cancer cells (Machado-Neto et al., 2014). In cancer cells, ANKHD1 is able to suppress p21 (Dhyani et al., 2015) and Stathmin activity (Machado-Neto et al., 2015). Despite the role of Mask or ANKHD1 in mitotic cells, the functions of Mask or ANKHD1 in post-mitotic cells, including neurons and muscle cells, are largely unknown. Our previous studies on Mask demonstrated that Mask regulates mitochondrial morphology (Zhu et al., 2015) and promotes autophagy (Zhu et al., 2017) in larval muscles. In the fly eye models for neurodegenerative diseases, overexpressing Mask in the photoreceptor cells mitigates degeneration caused by Tau overexpression (Zhu et al., 2017). In the present study, we show that Mask is required for the maintenance of a balanced MT stability in muscle and neurons, two post-mitotic cell types, and that Mask genetically interacts with Tau and Stathmin. Furthermore, the abundance of Jupiter, an MT-associated protein, is reversely related to Mask levels in the motor neuron axons. Knocking down *Jupiter* in the neurons partially suppresses the morphological defects caused by *mask* loss-of-function at the larval NMJs. Taken together, our studies demonstrate a novel function of Mask that can affect MT stability in post-mitotic cells.

## RESULTS

### Mask negatively regulates MT length in larval muscles

**Fig. 1.**
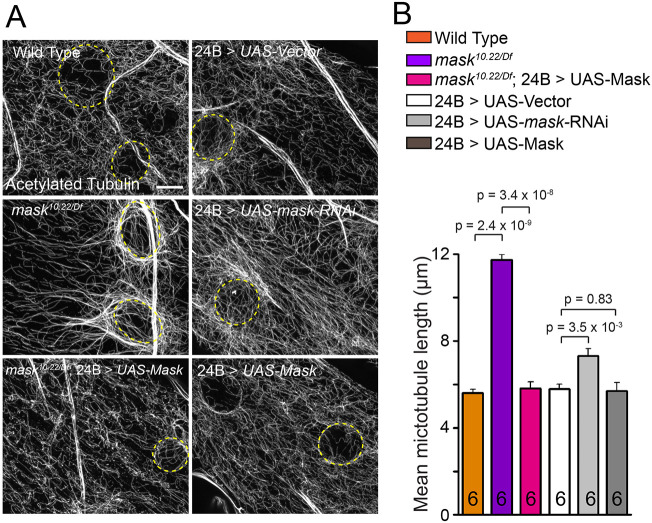
***mask* negatively affects MT stability in larval muscle.** (A) Confocal images (representative of three experiments) of MT in muscle 6 of wild type, *mask* null (*mask^10.22/Df^*), rescue of *mask* null with a 24B (muscle-specific)-Gal4-driven UAS-Mask transgene, 24B-Gal4-driven UAS-Vector, UAS-*mask* RNAi, or UAS-Mask. MTs were immunostained with an anti-acetylated-tubulin antibody. Yellow dashed lines denote the edge of muscle nuclei. Scale bar: 5 µm. (B) Quantification of average MT lengths.

Our previous studies of Mask demonstrated that overexpressing Mask ameliorates the degeneration of photoreceptors caused by overexpressing Tau in adult fly eyes (Zhu et al., 2017). This finding prompted us to explore the potential function of Mask in regulating the MT network, given that Tau is a well-studied MT-binding protein. In the larval body wall muscles, we found that the MTs visualized by either α-tubulin or acetylated tubulin show longer apparent length in the *mask* null mutants (*mask^10.22/Df^*) than in the wild-type larvae (Fig. 1; Fig. S1A). Further quantification of the stable MT polymers containing acetylated tubulin showed that their length in the *mask* null mutant muscles is nearly doubled compared to wild-type animals (Fig. 1). The *mask* mutant phenotype can be fully rescued by introducing the UAS-Mask transgene back to the *mask* mutant larval muscles (Fig. 1). In addition, muscle-specific knockdown of *mask* by RNAi moderately increased muscular MT length (Fig. 1), further confirming that Mask functions to restrain the length of the MT polymer in the muscle cells in a cell-autonomous manner. Overexpression of human Tau in fly larval muscles causes severe MT fragmentation (Xiong et al., 2013). Mask shows strong genetic interactions with the Tau overexpression in larval muscles – loss of *mask* function suppresses the Tau-induced MT breakdown (Fig. S2A,B), whereas upregulation of Mask enhances it.

**Fig. 2.**
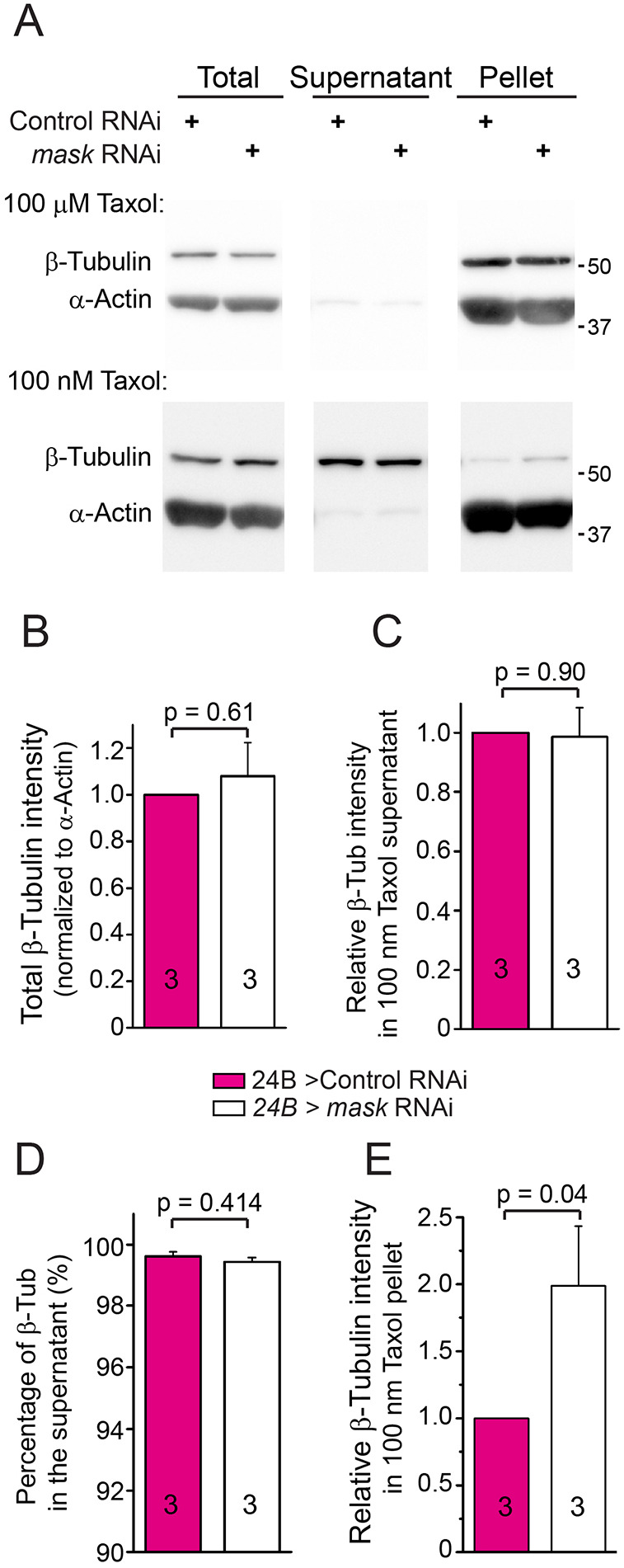
***mask* knockdown increases the sedimentation of MTs in fly larval muscles.** (A) Western analysis (representative of three experiments) of β-tubulin protein in total lysate or ultracentrifugal fractions (supernatant and pellet) in larval muscle lysates from control or *mask* knockdown (24B-Gal4-driven *mask* RNAi) animals. Lysates were treated with either 100 µm or 100 nm Taxol before ultracentrifugation. Anti-β-tubulin and anti-α-actin blots were performed for the western analysis. (B) Quantification of the total tubulin levels in the tissue lysates. The levels of tubulin were normalized to the levels of total α-actin in the same sample. (C) Quantification of the tubulin levels in the supernatant after centrifugation. The levels of tubulin were normalized to the levels of total actin in the same sample. (D) Quantification of the percentage of tubulin proteins that remained in the supernatant after centrifugation to total tubulin proteins present in the tissue lysate. (E) Quantification of the relative levels of tubulin fractionated in the pellet fraction in the lysate treated with 100 nm Taxol. The levels of tubulin in pellet were normalized to the levels of total tubulin in the same sample. The relative tubulin levels in the *mask* knockdown samples were then normalized to the relative tubulin levels in the control samples.

In addition to the morphological and genetic analyses, we performed biochemical analysis to determine the overall distribution of MT polymers in the cell lysates of control or *mask* knockdown larval muscles. We found that reducing Mask levels does not alter the total β-tubulin levels in muscle homogenates (Fig. 2A,B). In the presence of 100 μM Taxol [a potent concentration that promotes MT polymerization (Derry et al., 1995; Jordan and Wilson, 2004)], tubulin proteins in both control and mask *knockdown* lysates polymerize and fractionate exclusively in the pellet after ultracentrifugation (Fig. 2A). In the presence of 0.1 μM Taxol [a concentration that reserves a steady state of MT dynamic status (Derry et al., 1995; Jordan and Wilson, 2004)], the vast majority of tubulin proteins (∼99%) remain in the supernatant, while a small portion of tubulin fractionates in the pellet (Fig. 2C,D). Under these conditions, *mask* RNAi does not affect the overall levels of tubulin that remain in the supernatant (Fig. 2C), but the lysate contains more tubulin proteins that fractionate in the pellet after ultracentrifugation (Fig. 2A,E) compared to the control condition. Taken together, these results suggest that loss of Mask activity in muscles results in an altered MT network comprising MT polymers that are moderately more prone to sediment.

### Mask regulates presynaptic terminal growth in larval NMJs

**Fig. 3.**
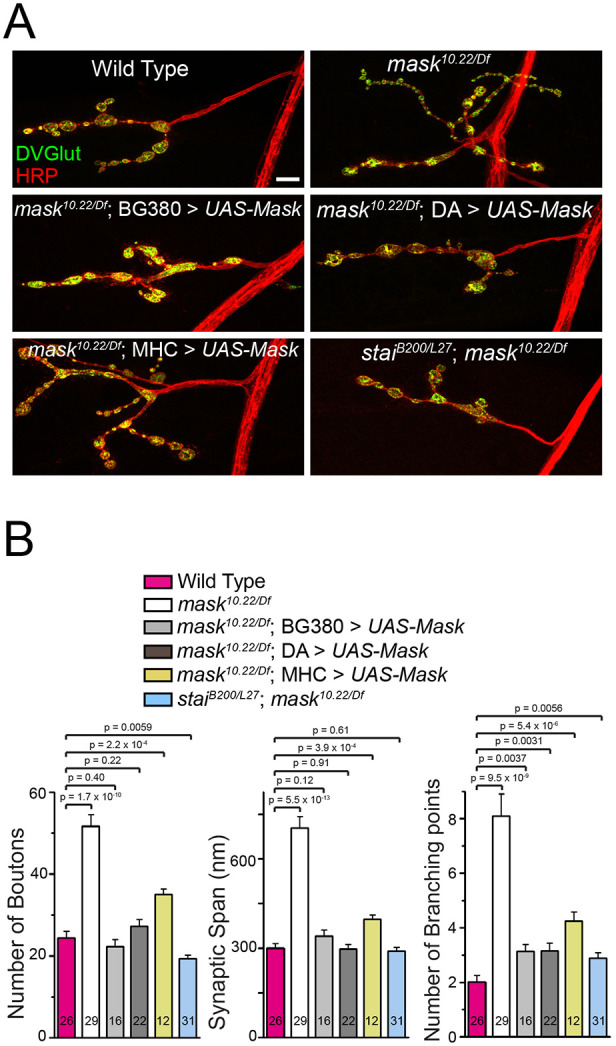
**Mask promotes normal NMJ terminal growth by affecting motor neuron MT stability.** (A) Confocal images (representative of three experiments) of NMJs of larval muscle 4 in wild type, *mask* null (*mask^10.22/Df^*), *mask* null rescued with a UAS-Mask transgene driven by pan-neuron (BG380), ubiquitous (DA) or muscle (MHC) Gal4 drivers, and *stathmin*;*mask* double mutant (*stai^B200/L27^*; *mask^10.22/Df^*). Scale bar: 10 µm. (B) Quantification of the number of boutons, synaptic span and branching points at the muscle 4 NMJs. Each data point was normalized to the size of the imaged muscle 4.

**Fig. 4.**
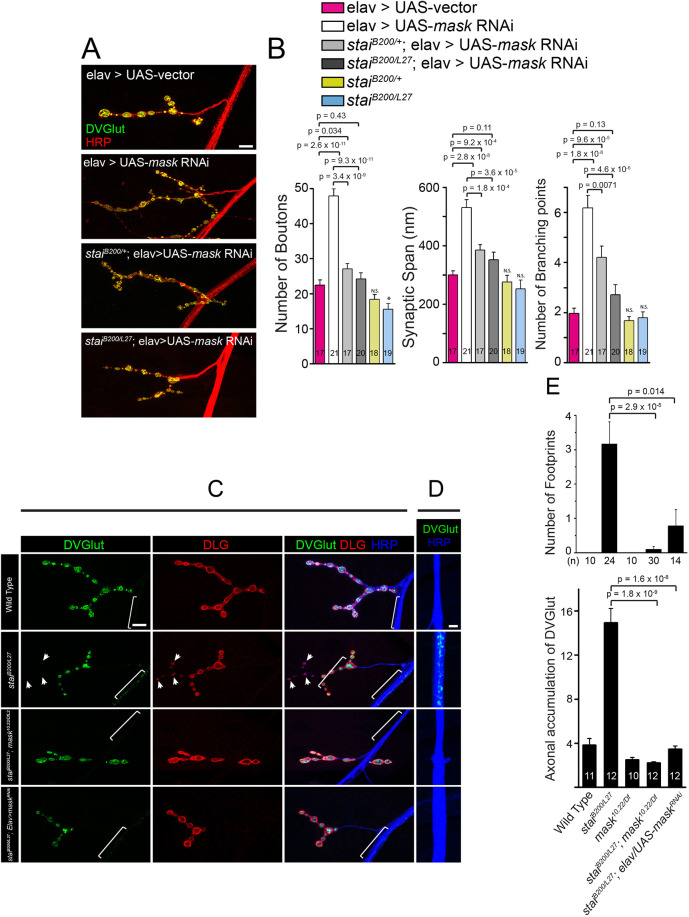
***mask* and *stai* genetically interact with each other.** (A,B) Loss of *stai* function suppresses the synaptic terminal over-expansion caused by *mask* loss of function in a dose-dependent manner. (A) Confocal images (representative of three experiments) of muscle 4 NMJs in larvae with Elav-driven UAS-vector, Elav-driven UAS-*mask* RNAi in wild-type background, *stai* heterozygous (*stai^B200/+^*) or *stai* homozygous (*stai^B200/L27^*) mutant backgrounds. NMJs were immunostained with anti-HRP (red) and anti-DVGlut (green) antibodies. Scale bar: 10 µm. (B) Quantification of the number of boutons, synaptic span and the number of branching points at the muscle 4 NMJs. Each data point was normalized to the size of the imaged muscle 4. (C–E) Loss of *mask* function in neurons suppresses *stai* mutant defects in NMJ stability and axonal transport. (C,D) Confocal images (representative of three experiments) of muscle 4 NMJs (C) and lateral nerve bundles (D) in wild type, *stai* (*stai^B200/L27^*), *stai*;*mask* double mutant (*stai^B200/L27^;mask^10.22/Df^*), or *stai* mutant with pan-neuronal expression of *mask* RNAi. Larval NMJs were immunostained with anti-DVGlut (green), anti-DLG (red), and anti-HRP (blue) antibodies. Arrowheads indicate synaptic boutons that contain postsynaptic DLG signals but lack presynaptic DVGlut staining (so-called ‘footprint’). Brackets highlight the lateral axons that exhibit residual DVGlut staining. Scale bars: 10 µm in C; 5 µm in D. (E) Quantification of the number of footprinting boutons in muscle 4 NMJs at segments A3 and A4, as well as abnormal accumulation of DVGlut in the axons. N.S., not significant; *P=0.04 (control vs *stai*^−/−^).

The prominent protective effects induced by overexpressing Mask in the photoreceptors (Zhu et al., 2017) also prompted us to analyze the neuronal functions of *mask*. Mask is expressed in the neurons and is ubiquitously distributed in the cell bodies (Fig. S3). We found that the larval NMJs in the *mask* null mutants (*mask^10.22/Df^*) show expanded presynaptic terminal growth reflected by increased number of boutons, synaptic span and number of branching points (Fig. 3A,B). This morphological defect is mostly due to the loss of *mask* function in the presynaptic motor neurons, as only pan-neuronal or ubiquitous expression of UAS-Mask completely rescues the NMJ terminal overgrowth phenotypes, whereas muscle (postsynaptic) expression of Mask could only moderately restore the NMJ morphology (Fig. 3A,B). Furthermore, neuronal knockdown of *mask* using *mask* RNAi causes similar NMJ expansions as observed in the *mask* genetic mutants (Fig. 4A,B).

### *mask* and *stathmin* genetically interact with each other to regulate the morphology and the structural stability of the larval NMJs

In addition to the motor neuron cell bodies, Mask can be detected in the axons but not at the NMJs (Fig. S3). In tissue homogenates of both larval brains and muscles, the majority of the Mask proteins fractionate in the pellet (containing debris, small membrane structures and insoluble proteins) after the first ultracentrifugation (100 000 ***g***), a distribution pattern that is largely non-overlapping with tubulins. However, after a 20 μM Taxol treatment that induces MT polymerization in the supernatant of the first ultracentrifugation, the soluble portion of the Mask proteins fractionate with MTs into the pellet after the second ultracentrifugation (180 000 ***g***) (Fig. S3D,E). Rae1, an evolutionarily conserved protein that binds to MTs and regulates mitosis and meiosis (Blower et al., 2005; Kraemer et al., 2001; Volpi et al., 2013), also precipitates with the Taxol-induced MTs (Fig. S3D–F). Results from these MT co-sedimentation assays suggest that a small portion of Mask proteins in the cells may be able to associate with MTs directly or indirectly.

Given that MT stability directly impacts the synaptic size and morphology (Chou et al., 2020), we then investigated whether the neuronal regulation of the presynaptic expansion by Mask is connected with its function in regulating the MT network. *stathmin* (*stai*) is a regulator for MT stability. It is highly expressed in the nervous system and is also detected in the early embryo and in the gonads (Lachkar et al., 2010; Ozon et al., 2002). Loss of *stai* causes severe destabilization of MT in motor neurons, resulting in reduced NMJ size and axonal transport defects, alongside a premature loss of presynaptic structure at the nerve terminals (known as the ‘footprint’ phenotype) (Duncan et al., 2013; Graf et al., 2011). Mammalian studies showed that ANKHD1, the human homolog of *mask*, regulates the activity of Stathmin 1 in leukemia cells (Machado-Neto et al., 2015), suggesting a possible link between the two genes in neurons. We found that the presynaptic NMJ expansion observed in *mask* mutant NMJs is completely suppressed in the *stai;mask* double mutants (Fig. 3A,B). Similarly, the NMJ expansion phenotype induced by neuronal knockdown (RNAi) of *mask* can also be partially suppressed by a heterozygous *stai* mutant, and completely suppressed by the homozygous *stai* mutant (Fig. 4A,B). In addition, *mask* loss of function can reciprocally suppress the neuronal defects caused by loss of function of *stai*. Both the ‘footprint’ phenotype and the axonal transport defect of *stai* mutants are partially suppressed by the neuronal knockdown of *mask* and completely suppressed by the *mask* null mutant (Fig. 4C–E), suggesting that the enhanced MT stability by *mask* loss of function can compensate for the impaired MT stability caused by loss of function of *stai*. Given that *mask* and *stai* exert opposite effects on MT stability, their strong genetic interactions further indicate that *mask* regulates synaptic morphology and formation at the larval NMJs via its impact on MT stability.

Previous studies on *stai* showed that *stai* negatively regulates tubulin levels in neurons (Duncan et al., 2013; Graf et al., 2011). To test whether *stai*- and *mask*-dependent regulations on MTs may converge on the control of tubulin expression, we examined the level of total α-tubulin in the larval brain. Neither loss of function nor overexpression of Mask caused any statistically significant changes in the overall protein levels of total α-tubulin, acetylated tubulin or tyrosinated tubulin in the larval CNS (Fig. S4A,B). However, in the axons of the motor neurons, loss of function of *mask* leads to an increase in acetylated tubulin and a decrease in tyrosinated tubulin levels, suggesting that Mask promotes an MT property that contains less stable but more labile MT (Fig. S4C,D) in the axons.

### Structure and function analysis of Mask for its action in modulating MT stability

**Fig. 5.**
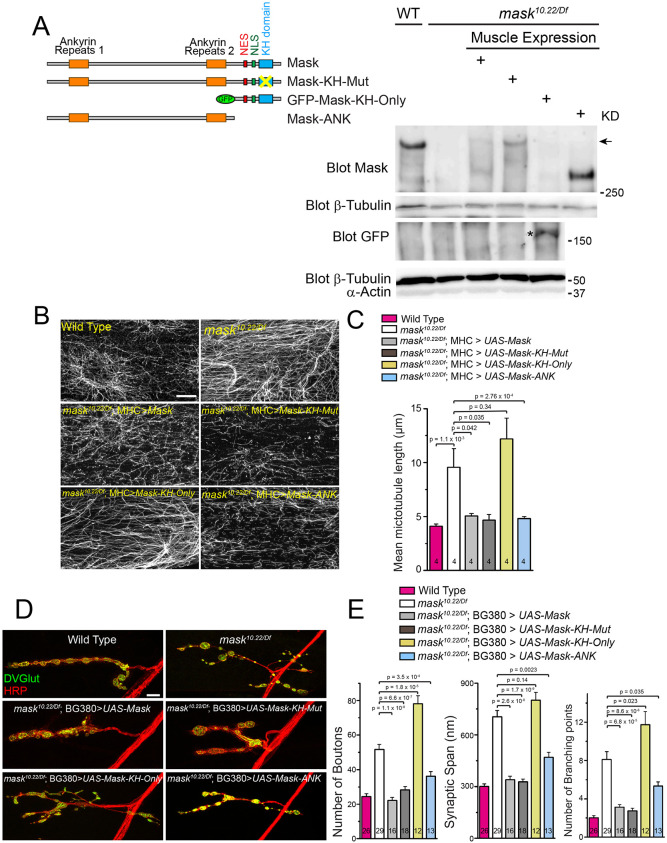
**Rescue experiments identify structural elements required for Mask's action in regulating MT stability.** (A) A schematic of wild-type and mutant UAS-Mask transgenes used in the rescue experiments, and western blots (representative of three experiments) showing muscle expression of endogenous Mask in wild type or each of the four UAS-Mask transgenes expressed in muscles in *mask* null mutants. Note that the anti-Mask antibody does not recognize GFP-Mask-KH-Only protein, indicating that the antigen of this antibody lies outside of the Mask-KH-Only region. Anti-GFP western blots were performed to show the expression of GFP-Mask-KH-Only (indicated by an asterisk). All transgenes were expressed with expected sizes. The arrow indicates the expected size of the endogenous Mask protein. KD, kDa. (B) Confocal images (representative of three experiments) of MT in muscle 6 of wild type, *mask* null (*mask^10.22/Df^*), and rescues of *mask* null with MHC-Gal4-driven UAS-Mask, UAS-Mask-KH-Mut, UAS-Mask-KH-Only, or UAS-Mask-ANK transgenes. MTs were immunostained with an anti-acetylated-tubulin antibody. Scale bar: 10 µm. (C) Quantification of MT lengths. (D) Representative confocal images of muscle 4 NMJs in wild type, *mask* null (*mask^10.22/Df^*), rescues of *mask* null with pan-neuron (BG380-Gal4) driven wild type or mutant/deletion UAS-Mask transgenes as shown in A. Scale bar: 10 µm. (E) Quantification of the number of boutons, synaptic span and the number of branching points at the muscle 4 NMJs. Each data point was normalized to the size of the imaged muscle 4.

Mask is a large protein containing multiple functional domains and motifs. In order to determine the domain requirement for its diverse functions, we generated a series of UAS-Mask transgenes carrying mutations or truncations of Mask functional domains. Recent studies showed that mutating the ‘GXXG’ loop to ‘GDDG’ in the KH minimal motif reduced the ability of the KH domain to bind RNAs (Hollingworth et al., 2012). The GXXG loop of Mask residing in amino acids 3053–3056 (GRGG) is completely conserved between fly Mask and human ANKHD1 (corresponding sequence 1710–1713). We generated a UAS-Mask transgene that carries a GRGG to GDDG mutation in their KH domain (named UAS-Mask-KH-Mut) as well as UAS-Mask deletion transgenes that lack either the N-terminal or the C-terminal portion of the protein (depicted in Fig. 5A). One resulting transgene contains the two ankyrin repeats clustered at the N-terminal portion of the protein (named Mask-ANK), and the other lacks the ankyrin repeats domain and contains only the NES, NLS and KH domains (named Mask-KH-Only).

All mutant *mask* transgenes were first validated for their expression with the predicted molecular weight (Fig. 5A) and then tested for their functionality for the abilities to rescue the MT-related loss of function phenotypes in larval muscles and the morphological defects at the larval NMJs. The results showed that the UAS-Mask-KH-Mut transgene rescues both the MT phenotype in muscles (Fig. 5B,C) and the defects of NMJ expansion at the larval NMJs in *mask* mutants (Fig. 5D,E) to a level comparable to the wild-type UAS-Mask transgene, suggesting that the function of the KH domain is not required for the activity of Mask to regulate MT stability or presynaptic terminal expansion. Meanwhile, the UAS-Mask-ANK, but not the UAS-Mask-KH-Only, transgene rescues the muscle MT length and NMJ overexpansion phenotypes in *mask* mutant larvae (Fig. 5B–E). Taken together, these results identify the N-terminal portion of the Mask protein that contains the ankyrin repeats domains as the necessary and sufficient structure element for its ability to affect MT stability.

### Mask inhibits the abundance of the MT-associated protein Jupiter in the motor neuron axons

**Fig. 6.**
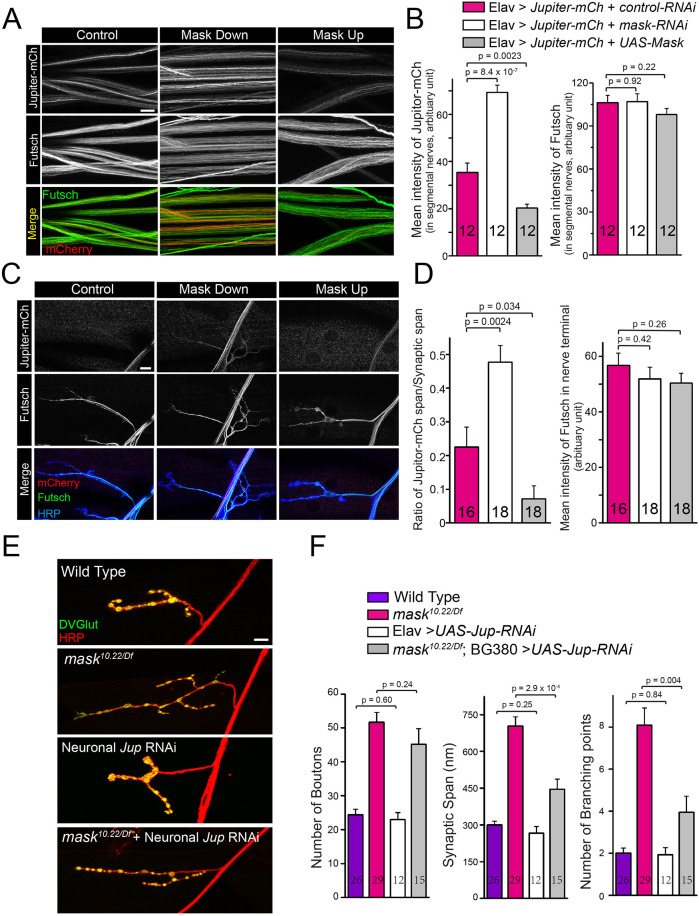
**Mask genetically interacts with Jupiter and regulates its distribution in the axons.** (A,C) Confocal images (representative of three experiments) of segmental nerves (A), or muscle 4 NMJs (C), immunostained with anti-mCherry (Red) and anti-Futsch (green) antibodies in 3rd-instar larvae of flies expressing Elav-driven UAS-Jupiter-mCherry together with UAS-control RNAi, UAS-*mask* RNAi or UAS-Mask. (B,D) Quantification of the mean intensity of mCherry and Futsch. (E) Confocal images (representative of three experiments) of muscle 4 NMJs in wild type, *mask* null (*mask^10.22/Df^*), Elav-Gal4-driven *Jupiter* RNAi (VDRC: KK116151), and BG380-Gal4-driven *Jupiter* RNAi in the *mask* null mutants. (F) Quantification of the number of boutons, synaptic span and the number of branching points at the muscle 4 NMJs. Each data point was normalized to the size of the muscle 4 that were imaged. Scale bars: 10 µm.

To further understand *mask*-mediated regulation of MT stability, we analyzed the potential interplays between Mask and two MT-associated proteins, Jupiter and Futsch, that have been implicated in the stabilization of MT (Karpova et al., 2006; Shi et al., 2019). Futsch is a neuronal-specific microtubule-associated protein (MAP) that distributes in the cell body, axons and synapses in the fly nervous system (Roos et al., 2000). Changes of Mask levels have no significant effects on Futsch intensity or distribution in the larval segmental nerves or NMJs (Fig. 6A–D). Jupiter is expressed in both neurons and non-neuronal tissues (Karpova et al., 2006). In motor neurons, it can be detected in the cell body and the axons, but not in the larval NMJs (Fig. S5).

Next, we tested whether Mask regulates Jupiter's subcellular distribution using a UAS-Jupiter-mCherry transgene. When expressed in the neurons, Jupiter-mCherry shows a distribution pattern similar to a Jupiter GFP trap line (Fig. 6A,B; Fig. S5). We found that *mask* loss of function substantially increases, whereas Mask overexpression reduces, the levels of the Jupiter-mCherry protein (Fig. 6A,B) as well as the endogenous Jupiter-GFP fusion protein (Fig. S5D,E) in the segmental nerves (axons). In addition, the increased abundance of Jupiter-mCherry caused by *mask* RNAi also leads to the extension of Jupiter-mCherry into the nerve terminal, whereas the reduced Jupiter-mCherry abundance caused by Mask overexpression leads to the retraction of Jupiter-mCherry further away from the nerve terminals. These effects are unlikely to result from changes in the overall Jupiter-mCherry protein levels, since neither *mask* RNAi nor Mask overexpression impacts the protein levels of Jupiter-mCherry (Fig. S6A). Taken together, these data suggest that Mask negatively regulates the abundance and distribution of the MT-associated protein Jupiter in the axons. It is worth noting that Jupiter-mCherry and Futsch appear to preferentially label distinct pools of MTs within the axons, which is more prominent in the segmental nerves of *mask*-knockdown animals (Fig. S6B). Although there is no prior evidence supporting the existence of distinct Jupiter-positive and Futsch-positive MT species that may be structurally and functionally different from one another, our data support the notion that Mask inhibits the decoration of MTs by Jupiter-mCherry but not Futsch.

We then tested the functional connections between *Jupiter* and *mask* by analyzing the genetic interactions between the two *mask* null mutants die at the late larval stage, and neuronal overexpression of Jupiter-mCherry in the *mask* null mutants induces lethality at the embryonic stage resulting in no *mask* mutant hatching from the eggs (*n*=523). Next, we identified a *Jupiter* RNAi line that can efficiently knock down *Jupiter* transcript levels to ∼33% in neurons (Fig. S6C,D). Neuronal knockdown of *Jupiter* alone does not affect synaptic terminal growth. However, in *mask* null mutants, it significantly suppresses the increased synaptic span and branching point phenotypes (Fig. 6E,F). Therefore, overexpressing Jupiter-mCherry in neurons enhances *mask* null lethality, whereas knocking down *Jupiter* in neurons partially suppresses the morphological defects caused by *mask* loss of function at the larval NMJs. The strong genetic interactions between *Jupiter* and *mask* not only provide a functional link between the two, but also provide a model in which Jupiter works downstream of Mask in the regulation of MT stability. Furthermore, these data provide new evidence supporting the role of Jupiter as an MT stabilizer.

## DISCUSSION

MTs in cells exist in both stable and labile states and undergo frequent growth and shrinkage as a result of dynamic instability (Mitchison and Kirschner, 1984). Proper maintenance of both the stable and dynamic MT pools is critical for normal cellular functions. Decades of studies have demonstrated that MT dynamics are tightly regulated by a number of mechanisms, including GTP:GDP ratio, post-translational modification of tubulins, and a vast array of MT-stabilizing, MT-polymerizing and depolymerizing, and MT-severing proteins. Our studies identify Mask as a novel regulator of MT stability that controls normal neuronal morphology during development and modulates MT stability in concert with factors such as *stai* that are related to human neuronal pathological conditions. Loss of *mask* resulted in elongated MTs in larval muscles. In neurons, *mask* loss of function causes presynaptic terminal over-expansion at the fly larval NMJs, possibly resulting from over-stabilization of MT.

The role of Mask in regulating MT stability is further supported by its genetic interactions with *stathmin* and *Jupiter*. Neuronal Stathmin family proteins are regulators of MT stability, and perturbation of Stathmin expression impacts neuronal development, plasticity and regeneration (Chauvin and Sobel, 2015). *Drosophila stathmin* mutations cause severely disrupted axonal transport and presynaptic nerve terminal growth and stabilization at the larval NMJs, probably resulting from impaired integrity of the MT network (Duncan et al., 2013; Graf et al., 2011). Knocking out mammalian STMN2, also known as SCG10, results in defects in axon outgrowth and regeneration (Klim et al., 2019). Loss of *mask* in neurons suppresses *stai*-induced axon transport and NMJ development phenotype in a dose-dependent manner, suggesting that Mask antagonizes the action of Stathmin in regulating MT stability. These data also suggested that manipulating Mask levels may have the ability to restore the stability and dynamics of the MT network that is disturbed under certain pathological conditions.

We previously reported that Mask regulates autophagy in fly larval muscles (Zhu et al., 2017). In line with the function of Mask, the levels of ectopic human Tau protein were significantly reduced when co-expressed with Mask, and it is substantially increased when co-expressed with *mask* RNAi (Fig. S2C,D). Moreover, Tau proteins in *mask*-knockdown muscles start to form aggregated puncta, possibly resulting from the excessively elevated levels of the Tau protein. Overexpressing Tau in the muscles causes the developing animals to die at the pupal stages, but simultaneously knocking down *mask* suppresses this lethality (X.T., unpublished data). Interestingly, these findings demonstrate that the formation of the Tau aggregates does not directly correlate with the toxicity and lethality caused by Tau overexpression in the muscles. It is most likely that the toxicity is primarily caused by the severe MT fragmentation induced by Tau expression. MT fragmentation induced by Tau does not seem to be correlative to Tau protein levels, as co-overexpressing Mask with Tau not only significantly reduces the levels of the exogenously expressed Tau protein, but also potently enhances MT fragmentation (Fig. S2A,B). Although these findings are not directly linked to this study on Mask, they suggest that dysregulation on Tau may cause defects in MT organization that is independent to the toxicity induced by Tau aggregates.

Our loss-of-function studies demonstrated that *mask* is required for normal MT organization in both neurons and muscles. Mask is a large scaffolding protein containing two ankyrin repeats at its N-terminus and a KH domain at its C-terminus. Ankyrin-repeat-containing proteins have been implicated in the regulation of MT dynamics. Two isoforms of *Drosophila* Ankyrin2, Ank2-L and Ank2-XL, regulate MT spacing in conjunction with Futsch and are involved with control of both axon caliber and transport (Stephan et al., 2015). The KH domain, on the other hand, may mediate the action of Mask in regulating RNA alternative splicing (Brooks et al., 2015) as well as transcription through the HIPPO pathway (Sansores-Garcia et al., 2013; Sidor et al., 2013). Our structure–function studies suggested that the two ankyrin repeats domains and not the KH domain are essential for the ability of Mask to regulate MT stability and synaptic morphology, providing not only knowledge for the structural basis of Mask-mediated regulation of MTs, but also additional supporting evidence of MT-dependent synaptic regulation by Mask. These data suggest that protein–protein interactions are essential for Mask to regulate MT stability, as opposed to directly binding to RNAs or DNAs. In consistence with this notion, Mask is not localized to the nuclei in either the muscles or the neurons (Fig. S3). Although the KH domain may not be essential for the action of Mask in regulating MT stability, it is required for Mask's activity in promoting autophagy. The KH domain mutation that impairs its RNA-binding capacity largely abolishes the ability of Mask to elevate autophagic flux in larval muscles (unpublished data). These results could indicate that different functions of Mask are mediated by separable structural elements and could be modulated independently.

Studies at the fly larval NMJ have discovered complex mechanisms regulating presynaptic development. A number of evolutionarily conserved pathways, such as the RPM-1/Hiw/Phr1-mediated ubiquitin pathways (Tian and Wu, 2013), the BMP signaling pathway (Bayat et al., 2011; Keshishian and Kim, 2004) and the Wnt signaling pathway (Koles and Budnik, 2012), are shown to control the normal presynaptic terminal size. The MT cytoskeleton also plays essential roles in sustaining synaptic morphology and function. Factors that directly regulate MT stability have been shown to impact axon transportation or synaptic structures. For example, Stathmin (Duncan et al., 2013; Graf et al., 2011), Ringer and Futsch (Shi et al., 2019), and dTACC (transforming acidic coiled coil) (Chou et al., 2020) regulate synaptic terminal growth at the fly NMJs, providing direct links that tie MT network dynamics to the control of the presynaptic size and morphology. Some signaling pathways control synaptic terminal growth by targeting MT stability, such as the *wg* signaling (Franco et al., 2004) and the FoxO pathway (Nechipurenko and Broihier, 2012). Our data demonstrate that loss of *mask* function stabilizes MT and promotes presynaptic expansion, providing additional evidence for a strong association between MT stability and presynaptic size.

Our imaging and genetic analyses of *mask* and *Jupiter* suggest that Mask negatively regulates MT stability by inhibiting the abundance of Jupiter-associated, but not Futsch-associated MTs. Jupiter is a known MAP and has been widely used as a marker for stable MTs (Karpova et al., 2006; Takeda et al., 2018), and it has been speculated that Jupiter promotes MT stabilization. Our analysis on Mask and Jupiter provides additional evidence to support an MT stabilizer function for Jupiter.

It is unclear how Mask inhibits MT localization of Jupiter in the axons. Mask is a protein that is distributed uniformly in the cytoplasm (Smith et al., 2002; Zhu et al., 2015) and in the axons of the motor neurons, and a small fraction of Mask protein may be associated with MTs (Fig. S3). Jupiter is an MT-associated protein that shows dynamic localization in the dividing cells in developing embryos (Karpova et al., 2006), and, in neurons, Jupiter exhibits a similar distribution pattern as Mask. Could these two proteins interact transiently and impact the ability of Jupiter to localize to the MTs? Or alternatively, is the binding of Jupiter to the MTs dictated by the property of the MTs in the axon, which is regulated by Mask? Loss of function of *mask* leads to increases in the acetylated tubulin and decrease in the tyrosinated tubulin in the axons (Fig. S4), which could consequently affect the stable association of Jupiter with the MTs. Moreover, the result that knocking down Jupiter can only partially suppress *mask* loss-of-function phenotypes indicates that Jupiter may not be the only downstream effector of Mask, and other factors may be required to work together with Jupiter and mediate the effects of Mask on MT stability. Identification of these factors holds the key to a better understanding of the molecular details for how Mask regulates MT stability.

## MATERIALS AND METHODS

### *Drosophila* strains, transgenes and genetics

Flies were maintained at 25°C on standard food. The following strains were used in this study: *mask^10.22^* (Smith et al., 2002), *mask^Df317^*, UAS-Jupiter-mCherry (Chabu and Doe, 2008), Jupiter GFP trap line (Karpova et al., 2006), BG380-Gal4 (neuron specific) (Budnik et al., 1996), Elav-Gal4 (neuron specific), MHC-Gal4 (muscle specific), 24B-GAL4 (muscle specific)*,* DA-Gal4 (ubiquitous), UAS-control-RNAi (P{TRiP.JF01147}), and UAS-*mask*-RNAi (P{TRiP.HMS01045}) from the Bloomington Drosophila Stock Center; and UAS-*Jupiter*-RNAi (KK116151) from the Vienna Drosophila Resource Center (VDRC). The full-length wild-type *mask* cDNA (Zhu et al., 2015) was used to generate mutant and truncated *mask* cDNA, including pUAST-Mask-KH-Mut, pUAST-GFP-Mask-KH-Only and pUAST-Mask-Ank. To generate the UAS-Mask-KH-Mut transgenes, a complementary primer pair containing the mutated coding sequence (GGAGACGATGGA, mutations underlined) was used to facilitate the replacement of the amino acid sequence (AA3053-3056) GRGG in the wild-type Mask with GDDG in the Mask-KH-Mut transgene. We used the QuikChange II XL site-directed mutagenesis kit (Agilent Technology, Santa Clara, CA, USA) to substitute wild-type sequence with the mutant sequence. The entire UAS-Mask-KH-Mut coding region was then sequenced to ensure no unintended mutations were introduced. To generate the UAS-Mask-ANK and UAS-Mask-KH-Only transgenes, the KpnI (8001) site was used to separate the Mask encoding region into an N- and a C-terminal fragment; each was then used to generate the pUAST-Mask-ANK and pUAST-Mask-KH-Only, respectively. All transgenic fly lines were generated by BestGene Inc. (Chino Hills, CA, USA).

### Generating the guinea pig anti-Rae1 antibody

*Drosophila* Rae1 coding cDNA was subcloned into the pET42 plasmid. Rae1 proteins expressed in *Escherichia coli* were purified and used as the antigen to generate the guinea pig anti-Rae1 antibody by Pocono Rabbit Farm & Laboratory (Canadensis, PA, USA). The specificity of the antibody was validated by western analysis (Fig. S3F).

### Western blots

Western blots were performed according to standard procedures. The following primary antibodies were used: mouse anti-β-tubulin (1:1000, E7) and mouse anti-α-actin (1:1000, JLA20) from Developmental Studies Hybridoma Bank; and rabbit anti-Mask (1:2000) (Smith et al., 2002), mouse anti-mCherry antibody (1:1000, 1C51, NBP1-96752 Novus Biologicals), guinea pig anti-Rae1 antibody (1:500) and rabbit anti-GFP (1:1000, A11122, Invitrogen). All secondary antibodies were used at 1:10,000. Data were collected using an LAS-3000 Luminescent Image Analyzer (Fujifilm) and quantified using Multi Gauge software (Fujifilm).

### MT cosedimentation assay

The assay was adapted from Hughes et al. (2008). A total of 50 wild-type larval brains, or 25 wild-type larval body walls, were dissected and snap frozen in liquid nitrogen. The frozen tissues were homogenized in 500 μl of Brinkley renaturing buffer [BRB80: 80 mM PIPES, pH 7.8, 1 mM EGTA, 1 mM MgCl_2_, 0.1% NP-40, 1 mM NaVO_4_, 0.05 mM MG132, 1 mM PMSF, protease inhibitor cocktail (P8340, Sigma, St. Louis, MO, USA)]. Extracts were clarified by an initial ultracentrifugation at 100,000 ***g*** at 4°C for 40 min (Beckman rotor TLA100.3). Clarified supernatant was transferred to fresh tubes, and GTP and dithiothreitol were each added to the supernatant to a final concentration of 1 mM and incubated at 25°C for 5 min. Taxol was then added to the lysate to reach the final concentration of 20 µM and incubated at 30°C for 30 min. The mixture was then layered onto a same volume of pre-warmed (30°C) cushion buffer (BRB buffer+10% sucrose, 20 µM Taxol and 1 mM GTP) before centrifuging at 180,000 ***g*** at 25–30°C for 30 min. The supernatant and the remaining sucrose cushion were removed. The pellet was washed once with BRB buffer (supplemented with 20 µM Taxol and 1 mM GTP) before resuspension in 1× SDS loading buffer.

### Immunocytochemistry

Third-instar larvae were dissected in ice-cold PBS and fixed in 4% paraformaldehyde for 30 min. The fixed tissues were stained following standard procedures. The primary antibodies used were: mouse anti-DLG, anti-Futsch and anti-β-tubulin antibodies from the Developmental Studies Hybridoma Bank; rabbit anti-DVGlut (Daniels et al., 2004), rabbit anti-GFP (A11122, Invitrogen) at 1:1000, rabbit anti-mCherry (632496, Clontech) at 1:1000, mouse anti-acetylated tubulin (T6793, Sigma) 1:1000, mouse anti-Tau (12-6400, Invitrogen) at 1:1000, Cy3-conjugated goat anti-HRP, and Cy5-conjugated goat anti-HRP. The following secondary antibodies (from Jackson ImmunoResearch) were used: Cy3-conjugated goat anti-rabbit IgG at 1:1000, Dylight-488-conjugated anti-mouse IgG at 1:1000, and Alexa-Fluor-647-conjugated goat anti-HRP at 1:1000.

### Confocal imaging and analysis

Single-layer or z-stack confocal images were captured on a Nikon (Tokyo, Japan) C1 confocal microscope. Images shown in the same figure were acquired using the same gain from samples that had been simultaneously fixed and stained. For quantification of MT length, z-stack confocal images of MTs in larval muscle 6 in segment A2 were obtained double-blinded, and IMARIS software (Bitplane, Inc.) was used to quantify average MT length in randomly selected muscle areas (see below for details).

The number of boutons and branching points on larval muscle 4 of segments A2–A3 were counted manually. Measurements of synaptic span (total length of NMJs on the muscle surface) as well as the bouton and branching point counts on the same muscle 4 were performed using NIS-Elements imaging software (Nikon). The synaptic expansions were measured using Nikon NIS-Elements software manual measurement tools. Muscle areas of each corresponding muscle 4 were measured using an eyepiece reticle – a crossline micrometer ruler – under a 20× objective, and the muscle area was used to normalize all three NMJ parameters.

The ratio of the nerve terminal distribution of Jupiter-mCherry at the NMJs was measured as the ratio of the length of detectable Jupiter-mCherry signal in the nerve terminal to the total length of the same nerve terminal analyzed.

### Quantification of MT length in fly larval muscles

MT length was analyzed using IMARIS 9.2.1 imaging software. The analysis was performed in a double-blinded manner. An 80 μm×80 μm (1024×1024 pixel resolution) confocal image for muscle 6 was used in the analysis, and a vertical step size of 0.15 μm was used to precisely capture the entire depth of muscle volume containing the MT network. From each image, two 30×30 μm areas of reasonable clarity were selected for quantification. For each randomly chosen area, 100±50 tracings were performed manually (assisted by IMARIS), with the total number of traces for each muscle sample averaging ∼250 filaments (see representative three-dimensional images in Fig. S1B). In order to ensure accurate MT tracing and end-to-end length measurements in three-dimensional space, it was necessary to regularly switch between the three-dimensional and layer-by-layer views of the imaged MT mesh. Additionally, the angle of the confocal image was often changed to allow for further confirmation that a single MT filament was being traced.

### Fractionation of tubulin

Fractionation of β-tubulin was performed as described by Xiong et al. with minor modifications (Xiong et al., 2013). Larval muscles of 20 larvae from each genotype were dissected in PBS at room temperature (RT). These muscles were immediately homogenized in 300 µl of lysis buffer (150 mM KCl, 2 mM MgCl_2_, 50 mM Tris, pH 7.5, 2 mM EGTA, 2% glycerol, 0.125% Triton X-100, protease inhibitor cocktail) containing either 100 μM or 100 nM Taxol. After incubating for 10 min at RT, the homogenates were centrifuged at 1500 ***g*** for 5 min at RT to remove cellular debris. A small aliquot of the homogenate of each sample was collected to analyze the total β-tubulin level. The remainder was ultracentrifuged at 100,000 ***g*** for 30 min. After ultracentrifugation, supernatant and pellet were separated and analyzed by SDS-PAGE and western blotting.

### Statistical analysis

Statistical analysis was performed, and graphs were generated using Origin (OriginLab, Northampton, MA). Each data set was tested for normal distribution and then compared with other samples in the group (more than two) using one-way ANOVA followed by post-hoc analysis with Tukey’s test, or with the other sample in a group of two using *t*-test. All bar diagrams show mean±s.e.m. The *n* numbers of each statistical analysis are indicated in each graph. See Table S1 for a detailed description of *n* numbers and *P*-values for each figure.

## Supplementary Material

Supplementary information

Reviewer comments
