# Supplementary material for: Mask, the Drosophila ankyrin repeat and KH domain-containing protein, affects microtubule stability
Source: J Cell Sci. 2021 Oct 22;134(20):jcs258512. doi: 10.1242/jcs.258512 (PMC8572007; doi:10.1242/jcs.258512)
Supplement: Supplementary information [file joces-134-258512-s1.pdf]

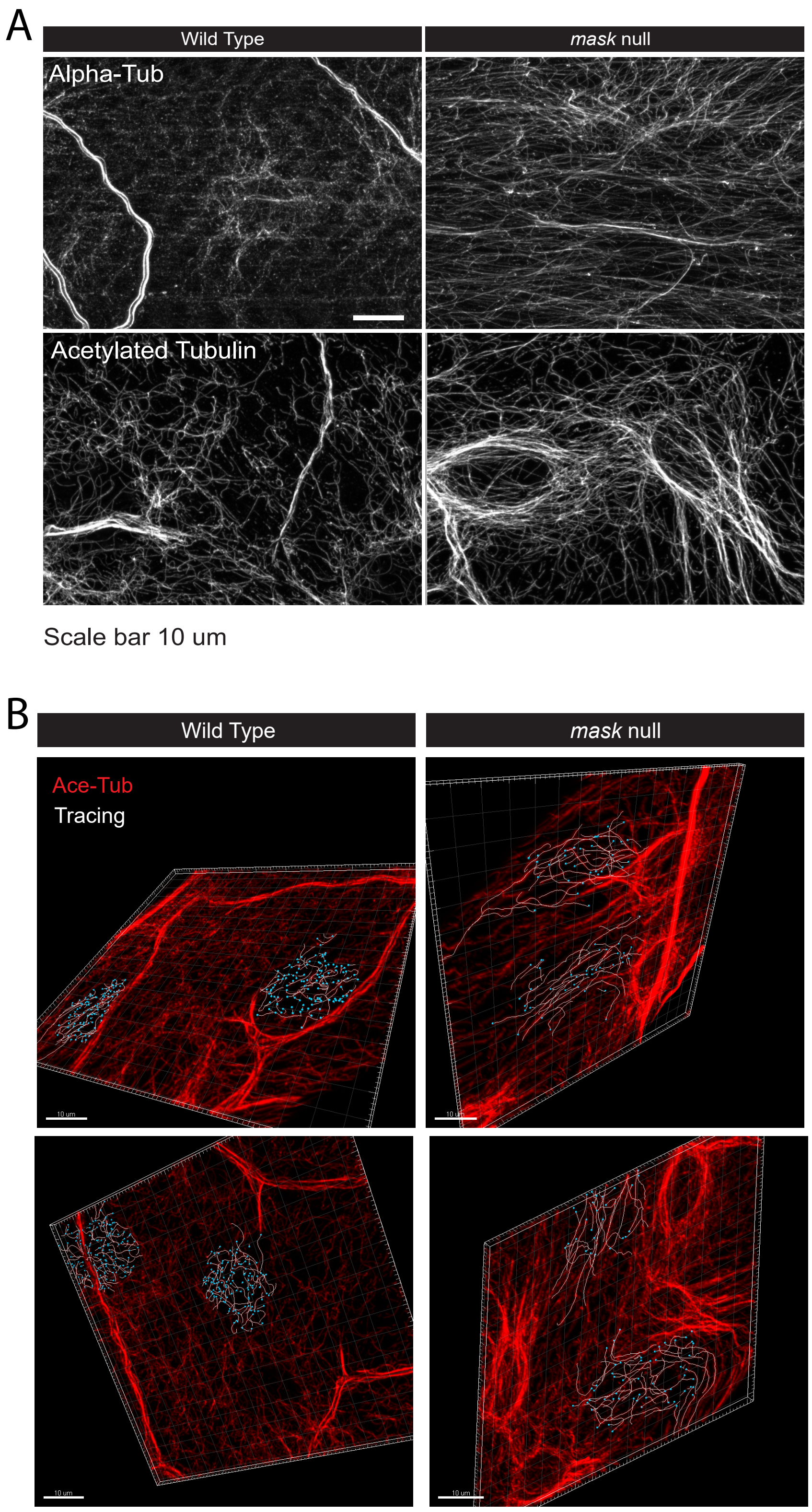

Fig. S1. Loss of Function of Mask results in elongated MT polymers in the larval muscle .

(**A**) Representative confocal images of MTs in muscle 6 are labeled with antibodies recognizing total α-tubulin or acetylated-tubulin. In the wild type larval muscles (left), the apparent length of the MTs is shorter than the MTs in *mask* mutants (right). Scale bar: 10 µm. (**B**) Representative Illustrations of MT length measurement in the larval muscles. Representative 3D images of MTs in muscle 6 in wild type (left) or *mask* mutant larvae (right). The stable portion of the MTs are labeled with anti-acetylated-tubulin. The 3D images are processed in the IMARIS imaging software, and the MTs in the areas were traced and highlighted in white for the quantification of their length. Scale bar: 10 µm.

Journal of Cell Science • Supplementary information

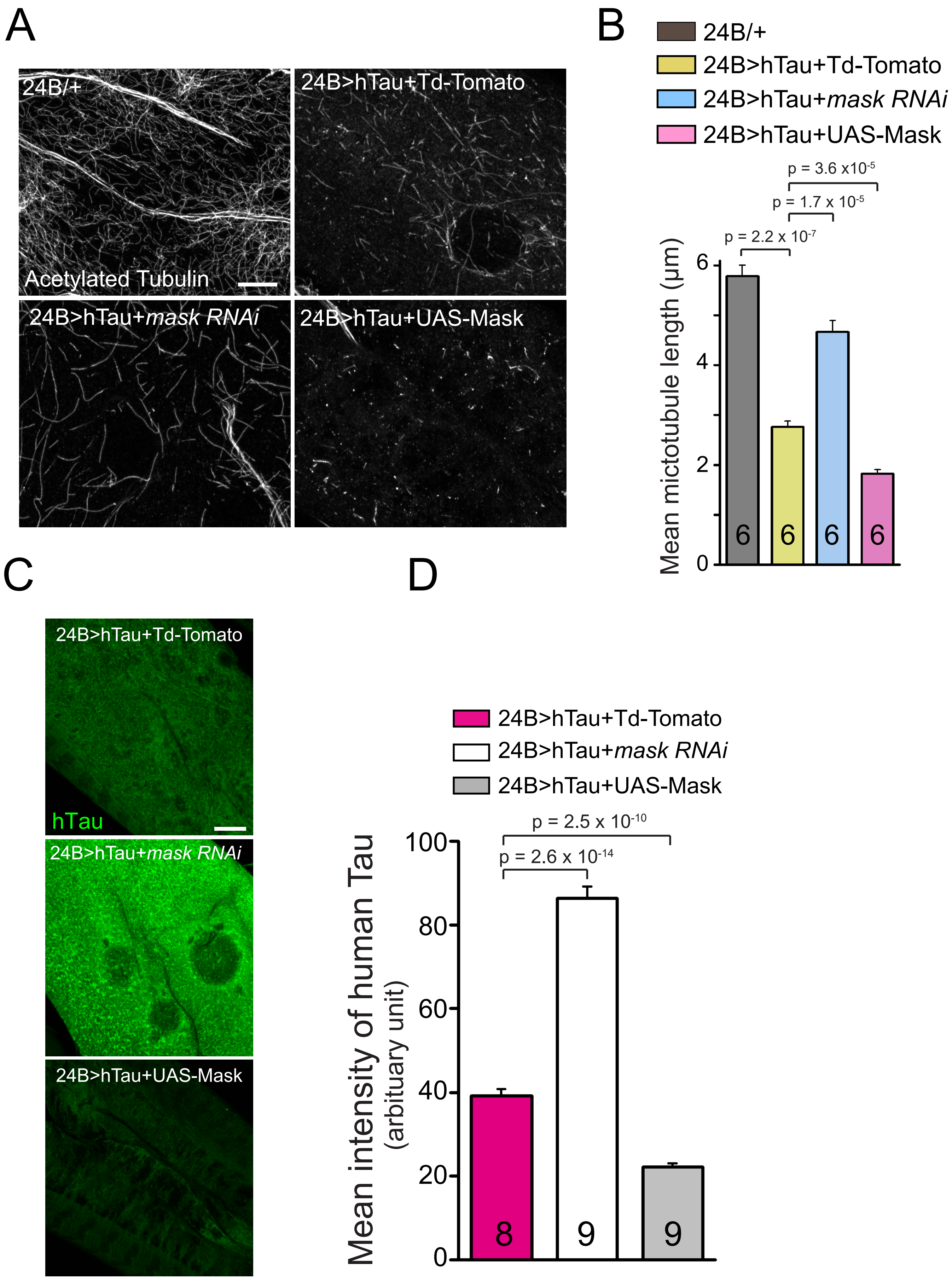

bar = 10 um

Journal of Cell Science • Supplementary information

Fig. S2. Mask Genetically interacts with Tau in the larval muscles.

(**A**) Representative confocal images of MT in muscle 6 of 24B-Gal4/+, 24B-Gal4-driven UAS-human Tau (hTau) together with UAS-Td-Tomato, UAS-*mask* RNAi, or UAS-Mask. MTs are immunostained with an anti-Acetylated Tubulin antibody. Scale bar: 5 µm. (**B**) Quantification of average MT lengths. (**C**) Representative confocal images of hTau expression in muscle 6 of 24B-Gal4-driven UAS-human Tau (hTau) with UAS-Td-Tomato, UAS-*mask* RNAi, or UAS-Mask. Human Tau proteins are immunostained with an anti-Tau antibody. Scale bar: 10 µm. (**D**) Quantification of mean intensity of hTau levels.

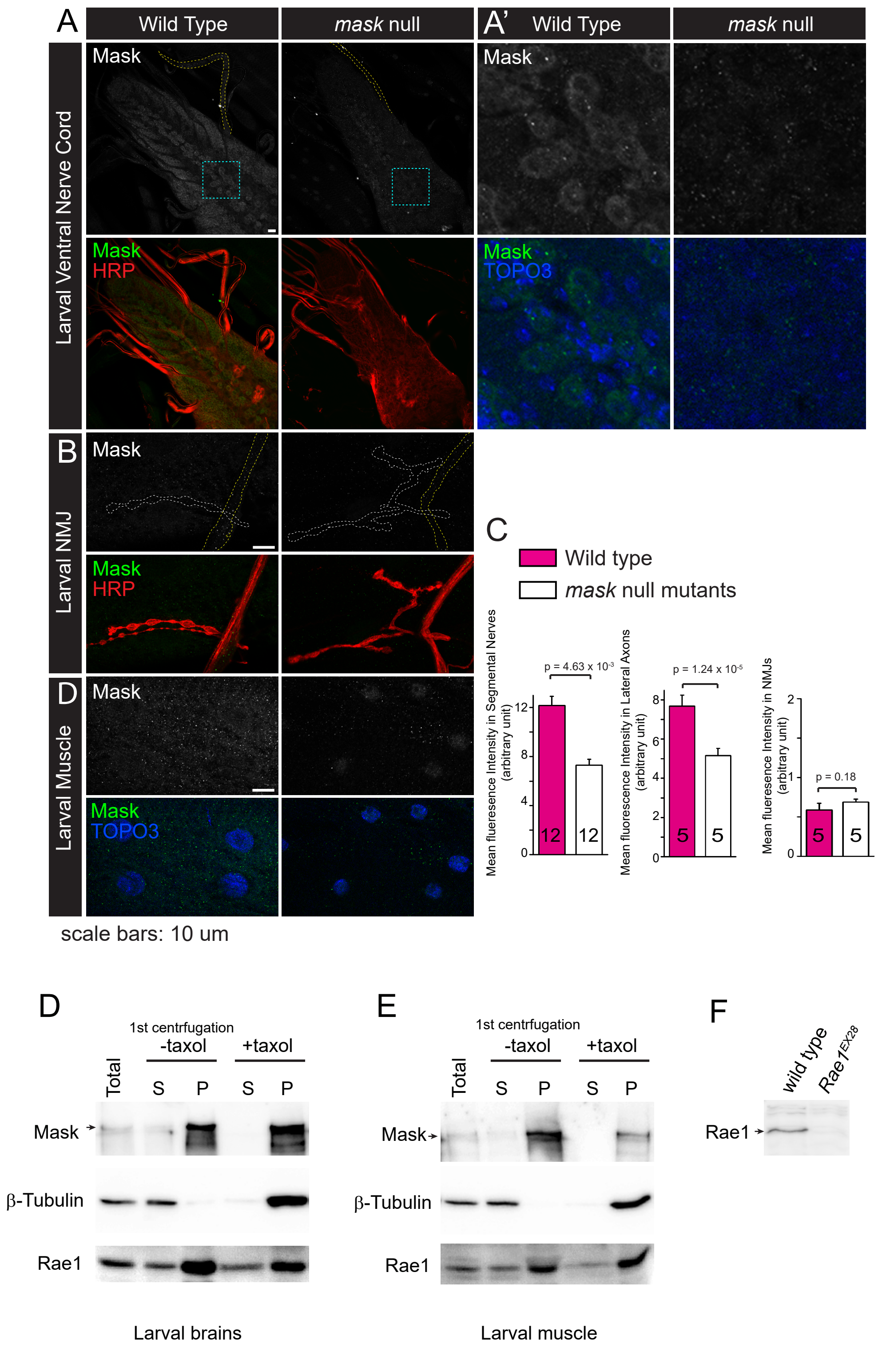

Fig. S3. Mask is distributed to the cell body and the axons in neurons, and can associate with taxol-induced MT polymers.

Representative confocal images of Mask immunofluorescence in wild type or *mask* protein null mutants. (**A**) Ventral nerve cord were immunostained with anti-Mask and anti-HRP antibodies. Mask protein is detected in the cell body and axons of the neurons (highlighted in dotted yellow lines). (A') Magnifications of areas in the ventral nerve cord (blue square). In the cell body, no

Journal of Cell Science • Supplementary information

overlap between Mask and TOPO3 (nuclei) can be detected, suggesting that Mask does not localized to the nuclei of the neurons. (**B**) Representative confocal images of NMJ synapses (white line) and lateral axons (yellow line) in wild type or *mask* protein null mutants were labeled with anti-Mask and anti-HRP antibodies. Mask protein can be detected in the lateral axons but not at the nerve terminals. (**C**) Quantification of the intensities of Mask immunofluorescence in wild type and *mask* null mutants. The fluorescence intensities of anti-Mask antibody detected in the segment nerve and the lateral axons in wild type larvae are higher than in the null mutants (background), suggesting that Mask protein is distributed to the axons. Mask fluorescence intensities at the NMJs show no differences between wild type and *mask* null mutants (background), suggesting that Mask protein is not localized to the synapses. (**D**) Representative muscle 6 in the 3rd instar larvae in wild type or *mask* null mutant animals were shown. Mask protein was detected with anti-Mask antibody. Comparisons between the wild type or *mask* null mutant larvae indicate that Mask is distributed ubiquitously in the cytoplasm of the muscle cells. Scale bar: 10 µm. (**D, E**) Western analysis of endogenous Mask, β-Tubulin and Rae1 proteins in the total lysate or ultracentrifugal fractions (supernatant and pellet) in larval brains (D) or muscles (E) . Lysates were subjected to an initial ultracentrifugation (100,000g) to separate the supernatant and the pellet (-taxol). The supernatant from the 1st centrifugation was treated with 20 µm taxol and then subjected to a second ultracentrifugation (180,000g) to precipitate the taxol-induced MTs and their associated protein complex (+taxol). (**F**) The specificity of the anti-Rae1 antibody was validated by western analysis of Rae1 protein in larval brain lysates from wild type or *Rae1* null mutant (*Rae1^{EX28}*) animals. The anti-Rae1 antibody recognizes Rae1 proteins in the wild type larval lysates but not the *Rae1* mutant lysates.

Journal of Cell Science • Supplementary information

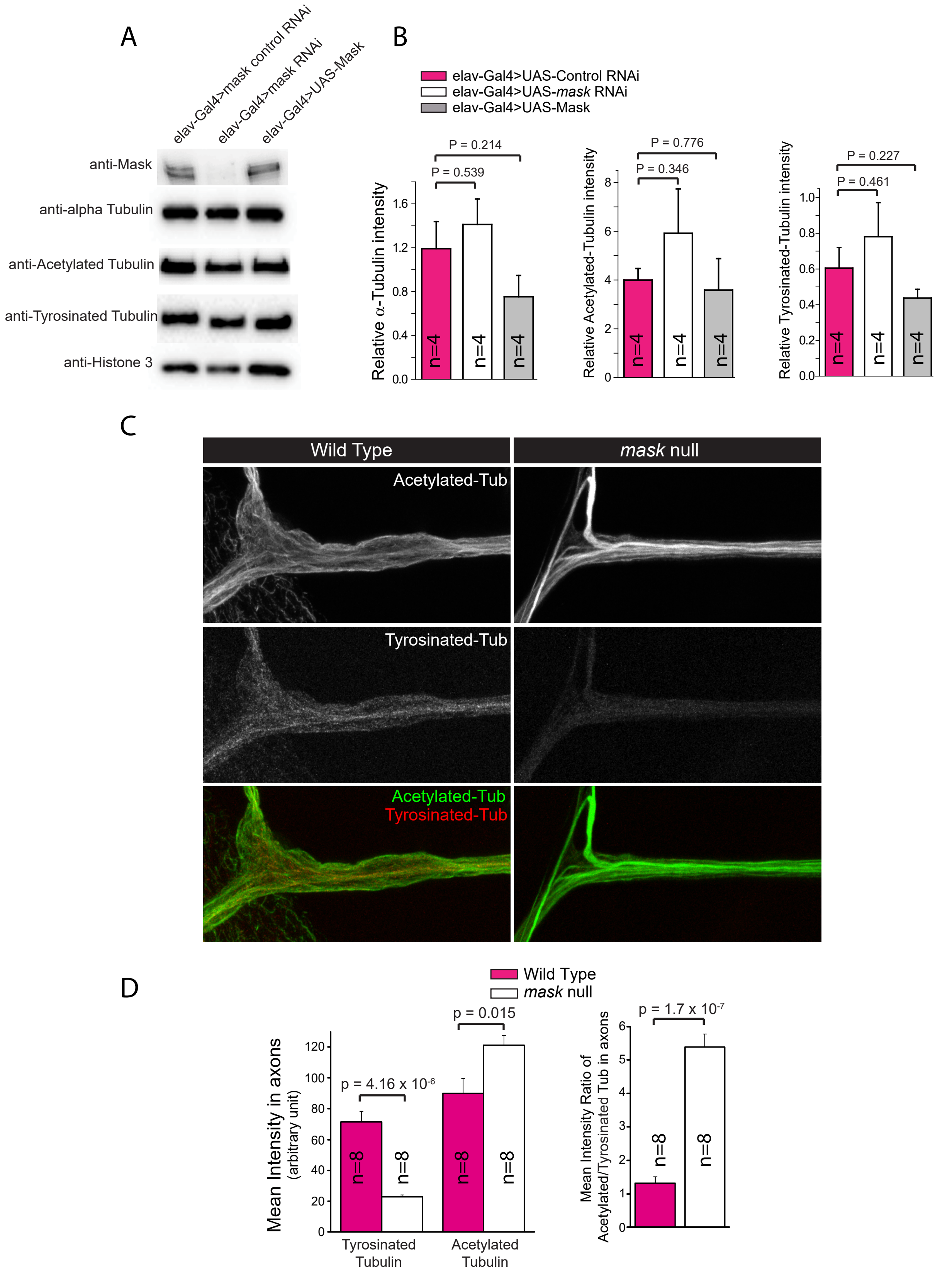

Fig. S4. Mask does not control the overall level of tubulin in fly brains, but it regulates the proportions of acetylated-tubulin or tyrosinated-tubulin in the axons.

(**A**) Representative western blot images of larval muscle lysates immunoblotted with anti-Mask, anti-α tubulin, anti-Ac-tubulin, anti-tyr-tub or anti-histone H3 antibodies. **B**) Quantification of expression levels normalized to histone H3. No statistically significant changes were detected for α-tubulin, Ac-tubulin or Tyr-tub MT in control, *mask* RNAi or Mask overexpression muscles. (**C**) Representative confocal images of lateral axons between muscle 4 and 5 in control, *mask* RNAi or overexpression larvae. Anti-acetylated-tubulin and anti-tyrosinated-tubulin antibodies were used to detect the stable and labile MT pools in the axons. (**D**) Quantification of mean intensities in axons.

Journal of Cell Science • Supplementary information

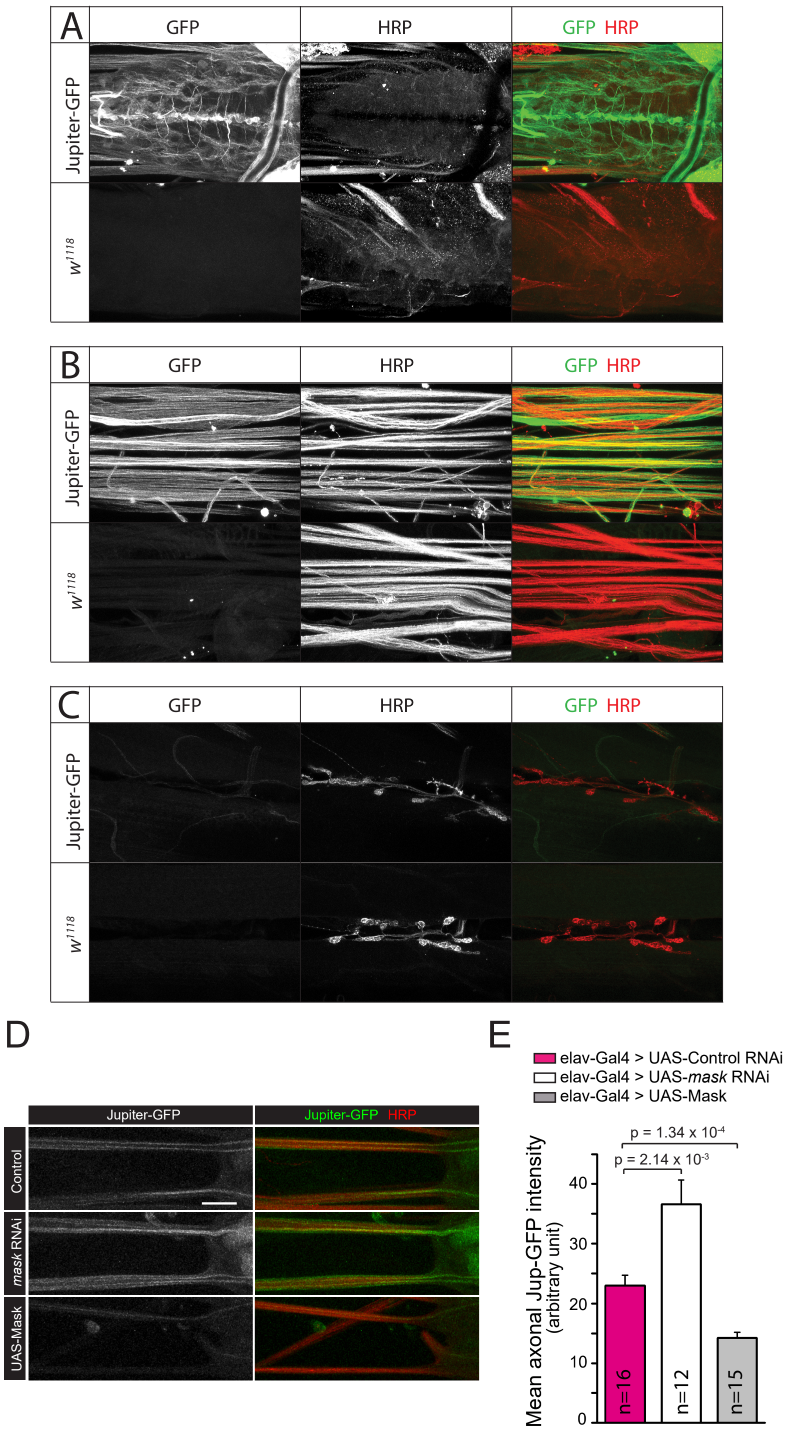

Fig. S5. Jupiter localizes to the cell body and axons but not the synapses in the fly larval motor neurons. A Jupiter GFP trap line was used to assess the expression and localization of the endogenous Jupiter protein, and the w^{1118} animals were used as background for GFP immunostaining signals. The larvae were fixed with 4% PFA briefly and then stained with an anti-GFP-488 antibody to visualize the Jupiter-GFP protein. The neuronal process were highlighted with an anti-HRP antibody. **(A)** Representative images of Jupiter-GFP protein in the larval CNS- the ventral nerve cord. Jupiter-GFP was detected in both the cell body and the axons in the CNS. **(B)** Representative images of larval segmental nerves. Jupiter-GFP protein show localization in the axons of the motor neurons. **(C)** Representative confocal images of NMJs at muscle 6/7. Jupiter-GFP protein does not localize to the nerve terminals of the NMJs. **(D)** Representative images of Jupiter-GFP protein in the segment nerves in 3rd instar larvae of flies expressing Elav-driven UAS-control RNAi, UAS-mask RNAi or UAS-*Mask*. **(E)** Quantifications of GFP intensity in the A8 segment nerves.

Journal of Cell Science • Supplementary information

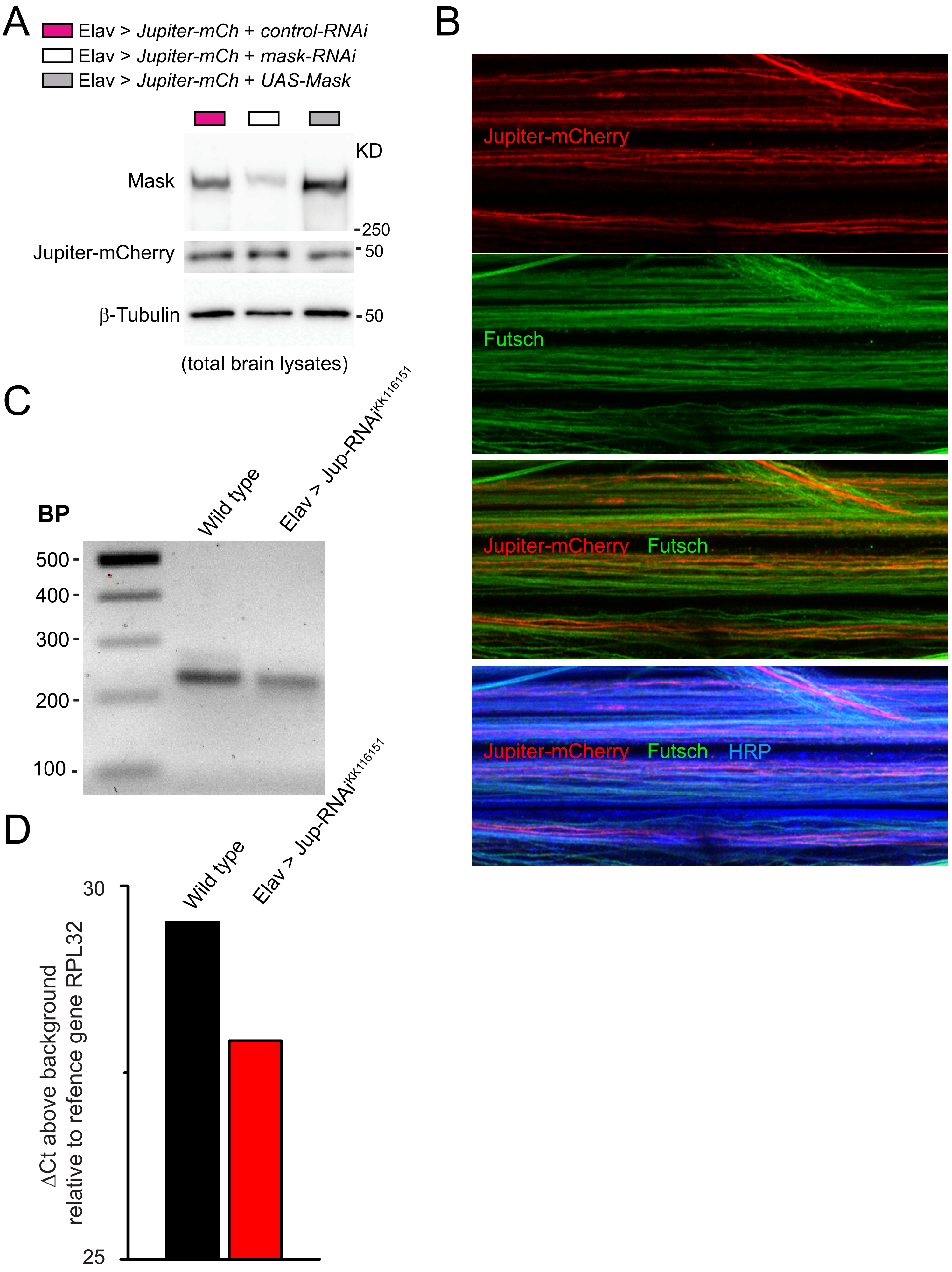
A
Elav > Jupiter-mCh + control-RNAi
Elav > Jupiter-mCh + mask-RNAi
Elav > Jupiter-mCh + UAS-Mask
KD
Mask
250
Jupiter-mCherry
50
β-Tubulin
50
(total brain lysates)
B
Jupiter-mCherry
Futsch
Jupiter-mCherry Futsch
Jupiter-mCherry Futsch HRP
C
BP
Wild type
Elav > Jup-RNAi[KK116151]
500
400
300
200
100
D
Wild type
Elav > Jup-RNAi[KK116151]
30
25
ΔCt above background
relative to refence gene RPL32

Fig. S6. Mask does not control Jupiter-mCherry expression level in the larval brain, and Jupiter-mCherry and Futsch preferentially associate with distinct pools of MTs.

(**A**) Western blot analysis of larval brains expressing Jupiter-mCherry together with UAS-control RNAi, UAS-*mask*-RNAi, or UAS-Mask. anti-Mask, anti-mCherry, and anti-β-Tubulin antibodies were used for immunoblot. (**B**) Representative confocal images of segmental nerves of 3rd instar larvae of Elav-driven Jupiter-mCherry and UAS-*mask*-RNAi. Notice the largely non-overlapping pattern of Jupiter-mCherry and Futsch. Scale bar: 10 μm. (**C, D**) Neuronal expression of Jup-RNAi line KK116151 effectively knocks down Jupiter mRNA levels. (**C**) A representative image of conventional RT-PCR result showing an expected ~230-bp *Jupiter* PCR fragment was amplified from wild type larval brains or brains expressing *UAS-Jup RNAiKK116151* (driven by elav-Gal4). (**D**) Quantitative RT-PCR shows reduction of Jupiter mRNA to ~33% (2^-ΔCt) compared to wild type control. The transcript of RPL32 was used as the internal control (reference gene) to calculate the ΔCt in control and RNAi samples. Note that the y axis values are expressed as log2 values relative to levels in wells containing no cDNA ("backbround"=Ct 40) followed by RPL32 normalization. To plot "ΔCt above background", the Ct from gene-specific primer for Jupiter was subtracted from the background Ct ("backbround"=Ct 40) and then normalized to RPL Ct above background. In this way, higher numbers=higher amounts of starting RNA.

Journal of Cell Science • Supplementary information

Table S1. Genotype, number of data samples and additional statistics

Figure s	Genotype	n	Additional P value (significance) not presented on the figures
Fig. 1A,B	w^{1118}	6 (Total of 6 larvae were used. In each sample, 2 random areas in muscle 6 of A2 were selected for MT measurement. The average length from these areas was used to reflect the MT length for each animal and for statistics analysis.)	
Fig. 1A,B	$Mask^{10.22/Df317}$	6 (Same)	
Fig. 1A,B	*24B-Gal4, mask*$^{10.22}$*/UAS-Mask, mask*^{DF317}	6 (Same)	
Fig. 1A,B	*24B-Gal4/*UAS-Vector	6 (Same)	
Fig. 1A,B	*24B-Gal4/*UAS-*mask-*RNAi	6 (Same)	
Fig. 1A,B	*24B-Gal4/*UAS-Mask	6 (Same)	
Fig. 2A,B	*24B-Gal4/UAS-control-RNAi*	3 (Three sets of experiments were done. In each set of experiment, body wall tissue of 20 larval were combined for the analysis.)	
Fig. 2A,B	*24B-Gal4/UAS-mask-RNAi*	3 (same)	
Fig. 3A,B	w^{1118}	26 (NMJs of muscle 4 in A2 and A3 in each larva were used in the analysis, i.e. up to 4 units may be counted in each animal.)	
Fig. 3A,B	$Mask^{10.22/Df317}$	29 (same)	
Fig. 3A,B	*BG380-Gal4; mask*$^{10.22}$*/UAS-Mask, mask*^{Df317}	16 (same)	
Fig. 3A,B	*DA-Gal4, mask*$^{10.22}$*/UAS-Mask, mask*^{Df317}	22 (same)	
Fig. 3A,B	*MHC-Gal4, mask*$^{10.22}$*/UAS-Mask, mask*^{Df317}	12 (same)	
Fig. 3A,B	$stai^{B200}$*/* $stai^{L27}$*; Mask*$^{10.22}$ */Mask*^{Df317}	31 (same)	

Journal of Cell Science • Supplementary information

Fig. 4A,B	*Elav-Gal4/UAS-vector*	17 (NMJs of muscle 4 in A2 and A3 in each larva were used in the analysis, i.e. up to 4 units may be counted in each animal.)	
Fig. 4A,B	*Elav-Gal4/UAS- mask-RNAi*	21 (same)	
Fig. 4A,B	$stai^{B200}$/+; *Elav-Gal4/UAS- mask-RNAi*	17 (same)	
Fig. 4A,B	$stai^{B200}$/ $stai^{L27}$; *Elav-Gal4/UAS- mask-RNAi*	20 (same)	
Fig. 4A,B	$stai^{B200}$/+	18 (same)	
Fig. 4A,B	$stai^{B200}$/ $stai^{L27}$	19 (same)	
Fig. 4C,D,E	w^{1118}	10 for "foot-print" (NMJs of muscle 4 in A4 in each larva were used in the analysis, i.e. up to 2 units may be counted in each animal.)<br>11 for DVGlut intensity (Lateral nerve of muscle 4 in A4 in each larva were used in the analysis, i.e. up to 2 units may be counted in each animal.)	
Fig. 4C,D,E	$stai^{B200/L27}$	24 for "foot-print" (same)<br>12 for DVGlut intensity (same)	
Fig. 4C,D,E	$Mask^{10.22/Df317}$	10 for "foot-print" (same)<br>10 for DVGlut intensity (same)	
Fig. 4C,D,E	$stai^{B200}$/ $stai^{L27}$; $Mask^{10.22}$ /$Mask^{Df317}$	30 for "foot-print" (same)<br>12 for DVGlut intensity (same)	
Fig. 4C,D,E	$stai^{B200}$/ $stai^{L27}$; *Elav-Gal4/UAS- mask-RNAi*	14 for "foot-print" (same)<br>12 for DVGlut intensity (same)	
Fig. 5B,C	w^{1118}	4 (Total of 4 larvae were used. In each sample, 2 random areas in muscle 6 of A2 were selected for MT measurement. The average length from these areas was used to reflect the MT length for each animal and for statistics analysis.)	P = 0.50<br>*MHC-Gal4,* $mask^{10.22}$/*UAS-Mask,* $mask^{Df317}$<br>*v.s.*<br>*MHC-Gal4,* $mask^{10.22}$/*UAS-Mask-ANK,* $mask^{Df317}$
Fig. 5B,C	$Mask^{10.22/Df317}$	4 (same)	

Journal of Cell Science • Supplementary information

Fig. 5B,C	*MHC-Gal4, mask*$^{10.22}$*/UAS-Mask, mask*^{Df317}	4 (same)	
Fig. 5B,C	*MHC-Gal4, mask*$^{10.22}$*/UAS-Mask-KH-Mut, mask*^{Df317}	4 (same)	
Fig. 5B,C	*MHC-Gal4, mask*$^{10.22}$*/UAS-GFP-Mask-KH-Only, mask*^{Df317}	4 (same)	
Fig. 5B,C	*MHC-Gal4, mask*$^{10.22}$*/UAS-Mask-ANK, mask*^{Df317}	12 (NMJs of muscle 4 in A2 and A3 in each larva were used in the analysis, i.e. up to 4 units may be counted in each animal.)	
Fig. 5D,E	*w*^{1118}	26 (same)	P = 1.1x10^{-4}, number of boutons;<br>P = 0.0011, synaptic span;<br>P = 8.8x10^{-5}, Number of branching points;<br><br>*BG380-Gal4; mask*$^{10.22}$*/UAS-Mask, mask*^{DF317}<br>*v.s.*<br>*BG380-Gal4; mask*$^{10.22}$*/UAS-Mask-ANK, mask*^{Df317}
Fig. 5D,E	*Mask*$^{10.22/Df317}$	29 (same)	
Fig. 5D,E	*BG380-Gal4; mask*$^{10.22}$*/UAS-Mask, mask*^{Df317}	16 (same)	
Fig. 5D,E	*BG380-Gal4; mask*$^{10.22}$*/UAS-Mask-KH-Mut, mask*^{Df317}	18 (same)	
Fig. 5D,E	*BG380-Gal4; mask*$^{10.22}$*/UAS-GFP-Mask-Only, mask*^{Df317}	12 (same)	
Fig. 5D,E	*BG380-Gal4; mask*$^{10.22}$*/UAS-Mask-ANK, mask*^{Df317}	13 (same)	
Fig. 6A,B	*Elav-Gal4;UAS-Jupiter-mCh/+; UAS-control-RNAi/+*	12 (areas in segment nerves that exit ventral nerve cord were analyzed. Two units from each larva were used.)	
Fig. 6A,B	*Elav-Gal4;UAS-Jupiter-mCh/+; UAS-mask-RNAi/+*	12 (same)	
Fig. 6A,B	*Elav-Gal4;UAS-Jupiter-mCh/+; UAS-Mask/+*	12 (same)	
Fig. 6C,D	*Elav-Gal4;UAS-Jupiter-mCh/+; UAS-control-RNAi/+*	16 (NMJs of muscle 4 in A2 and A3 in each larva were used in the analysis, i.e. up to	

Journal of Cell Science • Supplementary information

		4 units may be counted in each animal.)	
Fig. 6C,D	*Elav-Gal4;UAS-Jupiter-mCh/+; UAS-mask-RNAi/+*	18 (same)	
Fig. 6C,D	*Elav-Gal4;UAS-Jupiter-mCh/+; UAS-Mask/+*	18 (same)	
Fig. 6E,F	*w^{1118}*	26 (NMJs of muscle 4 in A2 and A3 in each larva were used in the analysis, i.e. up to 4 units may be counted in each animal.)	
Fig. 6E,F	*$Mask^{10.22/Df317}$*	29 (NMJs of muscle 4 in A2 and A3 in each larva were used in the analysis, i.e. up to 4 units may be counted in each animal.)	
Fig. 6E,F	*Elav-Gal4/ UAS-Jupiter-RNAi*	12 (NMJs of muscle 4 in A2 or A3 in each larva were used in the analysis, i.e. up to 2 units may be counted in each animal.)	
Fig. 6E,F	*BG380-Gal4; $mask^{10.22}$/ UAS-Jupiter-RNAi, $mask^{DF317}$*	15 (NMJs of muscle 4 in A2 or A3 in each larva were used in the analysis, i.e. up to 2 units may be counted in each animal.)	

Journal of Cell Science • Supplementary information
