# Supplementary material for: Mask, the Drosophila ankyrin repeat and KH domain-containing protein, affects microtubule stability
Source: J Cell Sci. 2021 Oct 22;134(20):jcs258512. doi: 10.1242/jcs.258512 (PMC8572007; doi:10.1242/jcs.258512)
Supplement: Reviewer comments [file joces-134-258512_review_history.pdf]

Journal of Cell Science

Mask, the *Drosophila* ankyrin repeat and KH domain-containing protein, affects microtubule stability

Daniel Martinez, Mingwei Zhu, Jessie J. Guidry, Niles Majeste, Hui Mao, Sarah T. Yanofsky, Xiaolin Tian and Chunlai Wu
DOI: 10.1242/jcs.258512

Editor: Giampietro Schiavo

Review timeline

Original submission:	9 February 2021
Editorial decision:	18 March 2021
First revision received:	21 June 2021
Editorial decision:	22 July 2021
Second revision received:	12 September 2021
Accepted:	16 September 2021

Original submission

First decision letter

MS ID#: JOCES/2021/258512

MS TITLE: Mask, the Drosophila Ankyrin Repeat and KH domain-containing protein, regulates microtubule dynamics

AUTHORS: Daniel Martinez, Mingwei Zhu, Jessie J. Guidry, Niles Majeste, Hui Mao, Sarah T. Yanofsky, Xiaolin Tian, and Chunlai Wu
ARTICLE TYPE: Research Article

We have now reached a decision on the above manuscript.

To see the reviewers' reports and a copy of this decision letter, please go to: https://submit-jcs.biologists.org and click on the 'Manuscripts with Decisions' queue in the Author Area. (Corresponding author only has access to reviews.)

As you will see, the reviewers raise a number of substantial criticisms that prevent me from accepting the paper at this stage. They suggest, however, that a revised version might prove acceptable, if you can address their concerns. If you think that you can deal satisfactorily with the criticisms on revision, I would be pleased to see a revised manuscript. We would then return it to the reviewers.

We are aware that you may be experiencing disruption to the normal running of your lab that makes experimental revisions challenging. If it would be helpful, we encourage you to contact us to discuss your revision in greater detail. Please send us a point-by-point response indicating where you are able to address concerns raised (either experimentally or by changes to the text) and where you will not be able to do so within the normal timeframe of a revision. We will then provide further guidance. Please also note that we are happy to extend revision timeframes as necessary.

Please ensure that you clearly highlight all changes made in the revised manuscript. Please avoid using 'Tracked changes' in Word files as these are lost in PDF conversion.

© 2021. Published by The Company of Biologists under the terms of the Creative Commons Attribution License (https://creativecommons.org/licenses/by/4.0/).

I should be grateful if you would also provide a point-by-point response detailing how you have dealt with the points raised by the reviewers in the 'Response to Reviewers' box. Please attend to all of the reviewers' comments. If you do not agree with any of their criticisms or suggestions please explain clearly why this is so.

Reviewer 1

Advance summary and potential significance to field

In the manuscript "Mask, the Drosophila Ankyrin Repeat and KH domain-containing protein, regulates microtubule dynamics" Martinez et al. investigate potential effects of downregulating the Mask gene on microtubules in Drosophila larval muscles and motoneurons, using a combination of mostly genetic and immunohistochemical assays. They find effects on the structure of muscle microtubules and NMJ morphology and a genetic interaction with the microtubule regulator stathmin. Thus, Mask may be a microtubule regulator in postmitotic cell types.

Comments for the author

While some of the findings are interesting and could point to a specific role of Mask protein as a microtubule regulator, the manuscript suffers from a number of shortcomings, including sometimes inappropriate choice of assays, overinterpretation of results, and bad writing. I cannot recommend publication in its current form but it might be suitable for publication after a major overhaul.
Positive - the actub stainings and quantifications look credible, as does the rather extensive domain analysis. This also applies to the NMJ experiments and stai genetic interactions, the most interesting part of the manuscript.
Criticisms.
- Some of the assays are inappropriate and hence the results are often overinterpreted. For example, the authors extensively use immunofluorescence to address questions of microtubule dynamics.

Example 1. The authors use immunofluorescence of overexpressed EB1::GFP to draw conclusions about microtubule plus ends (Figure 3C). Such conclusions can only be drawn from EB1::GFP live imaging to be able to distinguish EB1 comets from aggregates. This experiment must be removed.

Example 2. The authors co-overexpress human Tau protein with Mask constructs in muscles and assess microtubule structure. Drosophila Tau is not expressed in muscle, therefore, the assay and the conclusions are highly artificial.

Example 3. Throughout the manuscript, the authors assess microtubule structure by staining against ac-alphatub. This modification is known to occur only on stable microtubules, but not on their more dynamic parts which cannot be seen in this way. The conclusions regarding microtubule length are therefore overinterpreted.
A conceptual shortcoming of the manuscript is that the authors do not address the mechanism underlying the effect of Mask on microtubules. Mask is a large adaptor protein that is known to interact with transcriptional regulators (a description of the Mask protein, altough highly important for readers to understand the study, is also completely missing from the introduction). Is the effect on microtubules therefore a reflection of a transcriptional defect, or does Mask interact directly with microtubules? this is not even discussed. It is not known whether Mask colocalizes with microtubules or whether it is in the nucleus. The mask antibody used by the authors is suitable for immunofluorescence. Was this not even tried?

The biochemical assay is interpreted as showing that microtubules are longer in mask LOF. That conclusion can obviously not be drawn from a Western Blot.
How can this potentially be brought into a publishable form?
- add a immunofluorescence experiment to show localization of Mask.
- Describe mask in the introduction.
- remove Figure 3 C,D.
- The Jupiter data are also not very credible (overexpressed protein - not clear if it is actually expressed in motoneurons).
- remove Figure 1 C, D.

© 2021. Published by The Company of Biologists under the terms of the Creative Commons Attribution License (https://creativecommons.org/licenses/by/4.0/).

- conclusions regarding MT dynamics should be drawn much more carefully. If you want hard data, do EB1::GFP live imaging and quantify. Or, at least stain with other MT antibodies to detect labile parts of microtubules as well.

This would result in a shorter, but much more solid manuscript. Addition of Mask IF and EB1::GFP live imaging would strengthen the manuscript additionally.
other points The RNAi listed as "control" in the materials section (JF01147) actually targets Mask, according to Flybase. This should hopefully be a mistake.

Reviewer 2

Advance summary and potential significance to field

In this manuscript Martinez and colleagues describe a novel role of Drosophila Mask, a large Ankyrin repeat and KH domain containing protein in formation of MT networks in muscles and synaptic terminals in motorneurons. Using targeted expression of mask, mask-RNAi and mask mutants, the authors describe a length increase of acetylated MTs in larval muscles upon mask loss of function. They also find that loss of mask leads to an increase in presynaptic terminal growth in larval motorneurons. They furthermore describe a functional relationship between mask and three MT regulators: They find that mask expression can modify toxic effects of Tau overexperession in muscles. They observe that mask genetically interacts with Stathmin and Jupiter for the formation of presynaptic terminals. And they find that mask affects Jupiter localisation. A comprehensive structure function analysis shows that the KH domain is dispensable for mask's role in muscle MT length and synaptic terminal formation.

Overall, the authors present interesting findings and a novel role for mask that had been associated with signalling and proliferation previously. Masks therefore provides a new interesting regulator of MTs in axons and this study provides interesting findings for future mechanistic insights. However, the authors conclude that mask regulates MT dynamics and stability by affecting MT length, but evidence for this is largely indirect or data are open to other interpretations. Therefore, I do not agree with all conclusions and a few experiments are needed as controls.

Comments for the author

The authors measure MT length in muscles, however, by staining acetylated tubulin they only visualise a subset of MTs. Are only acetylated MTs affected or all MTs? Staining with an antibody recognising all tubulin pools (e.g. DM1A) could answer this. Minor point: A supplementary Figure illustrating how this was measured in 3D would help to understand how MT length can be analysed in those complex muscle MT networks.

I am not convinced that the MT pulldown assay (Figure 2) is suitable to allow MT length comparisons between the conditions. A stronger band in the pellet fraction can indicates higher MT amounts, but statements on MT properties like length should not be possible. A way to measure actual MT length would be to fix the lysates on coverslips and use TIRF microscopy imaging individual MTs. Further experimental issues are: The authors did not pre-clear the samples to remove previously polymerised MTs before the 10 minutes polymerisation step. Especially when differences are mild, this could affect outcomes. Also, the authors should provide references for the two taxol concentrations they used to substantiate the claim that 100uM promotes polymerisation and 100nM leads to steady state MT dynamics.

Deducing MTs are longer in axons purely from a reduction of Eb1 comet numbers (Fig. 3C) is highly speculative. Tubulin stainings have not been done in motoneurons and synaptic terminal formation is not a direct readout for MT stability in axons. I would suggest to phrase interpretations more carefully.

Besides Mask having an impact of Mask on MT length I would suggest two alternative explanations that could be tested experimentally and discussed:
- 1) Does Mask effect overall tubulin levels? The stronger, less curved acetylated MT bundles in muscles could reflect an increase in overall tubulin levels. This increase in tubulin could potentially explain the rescue of toxicity of Tau overexpression in muscles. Furthermore, it was

© 2021. Published by The Company of Biologists under the terms of the Creative Commons Attribution License (https://creativecommons.org/licenses/by/4.0/).

shown that tubulin levels are decreased in stathmin mutants (Duncan et al., 2013, PLOS ONE). Therefore, genetic interaction between stai and mask could be due to their opposite impact on tubulin levels. If that's the case I would expect to see a genetic interaction between mask and tubulin (e.g. combining mask mutants/RNAi with heterozygous alpha-tub mutants).
- 2) Does Mask function affect specific MT pools? There is a clear effect on acetylated tubulin when mask levels are reduced. Are overall tubulin pools affected (see above)? The authors didn't stain for tubulin in motorneurons. Are acetylated or overall tubulin levels increased in this context as well? The difference in mask-dependent mislocalisation of Jupiter and Futsch could be due to changed MT composition.
It feels a bit like a missed opportunity that the experiments with the three MT regulators are not done consistently in the same system. Specifically, it would be interesting to see if tau localisation is affected by Mask in motorneurons, a context where Tau function is important (analog to the Jupiter stainings). This could be analysed either overexpressing hTau or using the endogenously tagged P{Wee-P.un}tau304 line. Similar to the genetic interaction studies with stai and Jupiter: Does loss or gain of Tau affect mask-dependent synaptic overgrowth?
There is no experimental evidence that Mask controls MT dynamics (e.g. MT polymerisation speeds/lifetimes). Therefore, this statement should be reworded.
Out of interest: Could the authors speculate how Masks regulates MTs? By binding MTs directly, e.g. do the Mask constructs localise along MTs? Could it sequester other MTBPs?

Minor points:
- Would a non-parametric test be more appropriate to be used for statistics rather than a T-test? Have the authors tested if the data are distributed normally?
- Figure 4A, B: stai+/- and stai-/- alone as controls are missing
- Figure S3: Control gene is missing in the gel (A). Please provide expression values relative to control in Fig. S3B.

Reviewer 3

Advance summary and potential significance to field

Overview This study of Mask gene function using the fly NMJ as a model complements the previous papers on Mask. The authors use looking at the cell biological and molecular functions of Mask relative to multiple cytoskeletal components known to be important in this system. The authors show that Mask is necessary and sufficient to regulate MT density and apparent length in larval muscle, in a way that synergizes with Tau over expression. The authors' findings indicate that the ANK domain of this conserved protein family contributes to MT regulation in both neurons and muscle, suggesting that Mask may play a role opposing Tau and upstream of Jupiter.

Comments for the author

Overall, this is an interesting and novel story, and the data documentation appears to be of good quality. There are a few substantive issues that should be addressed before publication, but in principle, the manuscript seems appropriate for the journal.

Major issues:
1A) The use of the word "Dynamics" in the title seems inappropriate, since the authors do not show live imaging to demonstrate a change in MT s dynamic instability behavior (see 1B) -- thus, the title should be modified or new data added.
1B) The authors measure MT length at the light level at a resolution where limited MT bunding cannot be distinguished from single MT polymers. While transmission electron microscopy, or super-resolution (e.g. STORM) techniques, would be required to distinguish a bundled chain of MTs from a single polymer of the same length, the authors should clarify that they are measuring apparent length or total polymer fuorescence intensity in the text to avoid misleading the reader.

2) Does suppression of TauOE in Muscle reflect two additive but opposite effects on MTs that simply compensate for each other because they manifest on MT length, or a direct and specific functional relationship between Tau and Mask. Perhaps one way to distinguish might be to use generic pharmacological compounds that either stabilize or destabilize MTs in living muscle pelts

© 2021. Published by The Company of Biologists under the terms of the Creative Commons Attribution License (https://creativecommons.org/licenses/by/4.0/).

(e.g. Taxol or Nocodozole) combined with perturbation in Mask and in vivo analysis of MTs. This could complement the biochemical MT extraction experiments shown in Fig 2.

3) EB1 “comets’ are shown in Figure 3 C, however, it is not clear that the puncate hotspots here reflect polymerizing ends, or aggregates of EB1-GFP (some appear much too large to represent MT plus ends. Moreover, there is much shaft binding of this EB1, which is only seen at levels of EB1 expression that perturb normal polymerization and dynamic instability. These data must be represented as time lapse movies to confirm that the puncta are moving at rates and directions consistent with MT polymerization. Otherwise, this panel should be removed and the text modified accordingly.

4) One issue here is that the authors move back and forth from muscle to neuronal phenotypes, as if they assume that the Mask mechanism will function similarly in these two tissues. The Stai and Jupiter assays are performed on peripheral axon bundles, and not in muscle, but the authors do not show or clearly cite data showing that Stai and Jupiter are exclusively neuronal. To put this on context, prior analysis of multiple conserved signaling pathways and cellular processes have often shown that molecules function in quite different ways in the two cell types. This leaves the reader a bit confused when the authors turn to the question of cell type specificity in the discussion. Either a complete set of parallel assays in neurons or muscle is needed, or very careful clarification in the text.

5) Overall, the n values for NMJ sample number are very low in some genotypes (Fig 1B and 4E) – the authors should point out how many structures are being counted in each biological sample.

Minor Issues: English Grammar/Spelling
The authors should carefully proofread before submitting the revision; examples are here with correction in parentheses:
122 Our previous studies of the putative scaffolding protein Mask demonstrated that overexpressing Mask ameliorate(s) the degeneration of photoreceptors
238 Mask inhibits the abundance of the MA(MT)-associated protein Jupiter in the axons

---

First revision

Author response to reviewers' comments

We thank the reviewers for the constructive suggestions that help us to improve our manuscript. We have carefully considered and responded to each critique and suggestion. Please see below for our responses to your critiques: our responses follow below each comment and are prefaced by “Author response.” Corresponding changes were not specifically highlighted in the manuscript text in the revised file due to extensive editing of the original manuscript. Instead, we included in a separate PDF file all track changes made from last submitted manuscript.

Thank you again for your consideration of our revised manuscript.

Reviewer 1

Criticisms.
- Some of the assays are inappropriate and hence the results are often overinterpreted. For example, the authors extensively use immunofluorescence to address questions of microtubule dynamics.
Example 1. The authors use immunofluorescence of overexpressed EB1::GFP to draw conclusions about microtubule plus ends (Figure 3C). Such conclusions can only be drawn from EB1::GFP live imaging to be able to distinguish EB1 comets from aggregates. This experiment must be removed.

Author response: The original Figure 3C is removed from the manuscript.

© 2021. Published by The Company of Biologists under the terms of the Creative Commons Attribution License (https://creativecommons.org/licenses/by/4.0/).

Example 2. The authors co-overexpress human Tau protein with Mask constructs in muscles and assess microtubule structure. Drosophila Tau is not expressed in muscle, therefore, the assay and the conclusions are highly artificial.

Author response: The data regarding genetic interaction between Tau and *mask* is removed from the Figure 1. The revised manuscript is more focused on genetic interactions between *mask* and *stai*. Although ectopic expression of human Tau in fly muscle was used to model the MT dysfunction in AD-related degeneration (Xiong et al., 2013), it is not a best relevant physiological condition. However, we believe the interaction between Mask and Tau under this condition is still informative, and such data is now presented in Fig. S2.

Example 3. Throughout the manuscript, the authors assess microtubule structure by staining against ac-alphatub. This modification is known to occur only on stable microtubules, but not on their more dynamic parts which cannot be seen in this way. The conclusions regarding microtubule length are therefore overinterpreted.

Author response: We have reworded our conclusion. We provided new data (Fig. S1) to show that, in *mask* null mutants, the muscular MT-network immunostained with DM1A (recognizes all α-tubulins) exhibits a morphological phenotype very similar to the MT-network immunostained with Acetylated-Tubulin.

A conceptual shortcoming of the manuscript is that the authors do not address the mechanism underlying the effect of Mask on microtubules. Mask is a large adaptor protein that is known to interact with transcriptional regulators (a description of the Mask protein, altough highly important for readers to understand the study, is also completely missing from the introduction). Is the effect on microtubules therefore a reflection of a transcriptional defect, or does Mask interact directly with microtubules? this is not even discussed. It is not known whether Mask colocalizes with microtubules or whether it is in the nucleus. The mask antibody used by the authors is suitable for immunofluorescence. Was this not even tried?

Author response: In the revised manuscript we showed that Mask is ubiquitously distributed at the cytoplasm in the postmitotic muscles and motor neuron cell bodies. It is also present in the axons although it is not clear whether it binds to the MTs there. Mask was not found at the synapses of the NMJs (new Fig. S3). These results are consistent with previously published data of Mask localization: Mask show low and ubiquitous expression in the cytosol of larval muscles{Zhu, 2015 #572}; Mask was largely found in the cytosol in the photoreceptors in the developing eye discs and in the cultured S2 cells{Smith, 2002 #737}. However, Mask was shown to shuttle in and out of the nuclei in dividing cells {Sansores-Garcia, 2013 #497}{Sidor, 2013 #495}.

The biochemical assay is interpreted as showing that microtubules are longer in mask LOF. That conclusion can obviously not be drawn from a Western Blot.

Author response: We reworded our conclusions in the revised manuscript. We stated that MTs fractionated to the pellet are larger in mass, instead of claiming that the MTs are longer. The amount of the MTs present in the pellet that can be quantified by Western Blot reflects the amount of MT polymers with larger mass present in the cells. Our data demonstrated that more MTs are found in the pellet fraction of the muscle lysate of *mask* loss of function larvae, indicating that these muscle cells contain more MTs that are larger in mass.

How can this potentially be brought into a publishable form?

- add a immunofluorescence experiment to show localization of Mask.

Author response: Analysis of Mask localization was performed and presented in the new Fig. S3 (see above).

- Describe mask in the introduction.

Author response: Description of Mask is included in the Introduction: Line 92-120.

© 2021. Published by The Company of Biologists under the terms of the Creative Commons Attribution License (https://creativecommons.org/licenses/by/4.0/).

- remove Figure 3 C,D.

Author response: Done

- The Jupiter data are also not very credible (overexpressed protein - not clear if it is actually expressed in motoneurons).

Author response: We have now included new analysis of Jupiter expression in the revised manuscript (Fig. S5). Using a previously characterized GFP-trap line of Jupiter {Karpova, 2006 #993}, we determined that Jupiter is expressed in the nervous system. Jupiter proteins are distributed in the cell body and can also be detected in the axons of the motor neurons but not at the synapses of the NMJs. These data validate that the neuronal expression of the UAS-mCherry-Jupiter transgene (Fig. 6) recapitulates the subcellular distribution of the endogenous Jupiter protein.

- remove Figure 1 C, D.

Author response: Figure 1 C.D was moved to Fig. S2, see above.

- conclusions regarding MT dynamics should be drawn much more carefully. If you want hard data, do EB1::GFP live imaging and quantify. Or, at least stain with other MT antibodies to detect labile parts of microtubules as well.

This would result in a shorter, but much more solid manuscript. Addition of Mask IF and EB1::GFP live imaging would strengthen the manuscript additionally.

Author response: The EB1::GFP results were removed. We performed additional analysis on mobile vs. stable pool of MTs using antibodies against Tubulins with specific post-translation modification. We found that, in the motor neuron axons, the intensity ratio of Acetylated-Tub (stable):Tyrosinated-Tub (mobile) is greatly enhanced in *mask* lof compared to control (Fig. S2).

other points
The RNAi listed as "control" in the materials section (JF01147) actually targets Mask, according to Flybase. This should hopefully be a mistake.

Author response: The JF01147 RNAi line was initially designed to target mask, however, expressing this RNAi line does not reduce the level of endogenous Mask protein, nor does it induce *mask* loss of function phenotypes. Therefore, we have been using this line as the control RNAi for *mask* knock down analysis. The characterization of this control RNAi line and *mask* RNAi line was published in our previous work{Zhu, 2015 #736}.

Reviewer 2 Comments for the Author:
The authors measure MT length in muscles, however, by staining acetylated tubulin they only visualise a subset of MTs. Are only acetylated MTs affected or all MTs? Staining with an antibody recognising all tubulin pools (e.g. DM1A) could answer this. Minor point: A supplementary Figure illustrating how this was measured in 3D would help to understand how MT length can be analysed in those complex muscle MT networks.

Author response: As the reviewer suggested, we immunostained muscle MTs with DM1A antibody. We found that *mask* null muscles exhibit longer MTs compared to wild type muscles (Fig. S1), consistent with our analysis with the anti-Acetylated-Tubulin antibody. We also assessed tubulin levels through Western Blot analysis, and no changes of the overall levels of tubulin, acetylated-tubulin or tyrosinated-tubulin were detected in larval brain lysates (new Fig. S4 AB). However, our new results of the immunofluorescent analysis on the motor neuron axons showed that the intensity ratio of Acetylated-Tub (stable)/Tyrosinated-Tub (mobile) is greatly enhanced in *mask* lof compared to the control (Fig. S4CD).

3D representations of the muscle microtubules and the quantification process were shown in the new Supplemental Figure 2. The quantification was double blinded and areas of manual tracing

© 2021. Published by The Company of Biologists under the terms of the Creative Commons Attribution License (https://creativecommons.org/licenses/by/4.0/).

were randomly chosen as described in the method section.

I am not convinced that the MT pulldown assay (Figure 2) is suitable to allow MT length comparisons between the conditions. A stronger band in the pellet fraction can indicates higher MT amounts, but statements on MT properties like length should not be possible. A way to measure actual MT length would be to fix the lysates on coverslips and use TIRF microscopy imaging individual MTs. Further experimental issues are: The authors did not pre-clear the samples to remove previously polymerised MTs before the 10 minutes polymerisation step. Especially when differences are mild, this could affect outcomes. Also, the authors should provide references for the two taxol concentrations they used to substantiate the claim that 100uM promotes polymerisation and 100nM leads to steady state MT dynamics.

Author response: We rephrased our conclusions in the revised manuscript. Instead of claiming that the MTs are longer, we stated that MTs fractionated to the pellet are larger in mass. Because structures that are larger in mass tend to fractionate into the pellet, the results presented in Figure 2 that more MTs are found in the pellet fraction would indicate that cell lysate of *mask* lof muscles contains moderately bigger portion of MTs that are larger in mass. This conclusion is drawn from the results of 100nM treatment.

The 100uM treatment was used to indicate that polymerization of tubulin induced by taxol is not affected by *mask* loss of function, and a pool of free tubulin exists in the cell lysate under the *mask* loss of function condition.

The differential effects of microtubule target reagents was previously reported{Derry, 1995 #1049} and reviewed{Jordan, 2004 #1050}, and both references were included in the revised manuscript.

Deducing MTs are longer in axons purely from a reduction of Eb1 comet numbers (Fig. 3C) is highly speculative. Tubulin stainings have not been done in motoneurons and synaptic terminal formation is not a direct readout for MT stability in axons. I would suggest to phrase interpretations more carefully.

Author response: In light of comments from all reviewers, we have removed the EB1-GFP data from the manuscript. We did performed IF on Tyrosinated-Tub and Acetylated-Tub in the motor neuron axons, and a loss of *mask* function increases the intensity of Ace-Tub and decreases the intensity of Tyr-Tub, suggesting that loss of function of *mask* results in increased pool of stabilized MTs and decreased labile pool of MTs in the axons. These results were presented as the new Fig. S4.

Besides Mask having an impact of Mask on MT length I would suggest two alternative explanations that could be tested experimentally and discussed:
-1) Does Mask effect overall tubulin levels? The stronger, less curved acetylated MT bundles in muscles could reflect an increase in overall tubulin levels. This increase in tubulin could potentially explain the rescue of toxicity of Tau overexpression in muscles. Furthermore, it was shown that tubulin levels are decreased in stathmin mutants (Duncan et al., 2013, PLOS ONE). Therefore, genetic interaction between stai and mask could be due to their opposite impact on tubulin levels. If that’s the case I would expect to see a genetic interaction between mask and tubulin (e.g. combining mask mutants/RNAi with heterozygous alpha-tub mutants).

Author response: Mask loss of function does not seem to increase β-Tubulin protein levels in larval muscles (Figure 2). We further determined that loss of function of *mask* has no effect in the overall level of α-tubulin in the larval CNS, see in the new Fig. S4, using DM1A (anti-α- Tubulin) immunoblot on the homogenate of larval CNS. Based on these results, we believe that regulation of tubulin levels is unlikely to be the convergent point between *mask* and Tau, or *mask* and *stai*.

-2) Does Mask function affect specific MT pools? There is a clear effect on acetylated tubulin when mask levels are reduced. Are overall tubulin pools affected (see above)? The authors didn’t stain for tubulin in motorneurons. Are acetylated or overall tubulin levels increased in this context as well? The difference in mask-dependent mislocalisation of Jupiter and Futsch could be due to changed MT composition.

© 2021. Published by The Company of Biologists under the terms of the Creative Commons Attribution License (https://creativecommons.org/licenses/by/4.0/).

Author response: We analyzed total Tubulin (anti-DM1A) as well as acetylated-Tubulin and tyrosinated-Tubulin in wild type and *mask* mutants (see above). In the larval CNS, the western analysis suggested that a loss of *mask* function does not affect the overall levels of total tubulin, stabilized MT pool (ac-tub) or mobile MT pool. However, in the motor neuron axons, the intensity of ac-tub increases and tyr-tub decrease (new Fig. S4CD).

We agree that the altered MT composition may be a direct contributing factor to *mask*-dependent regulation of the distribution of Jupiter in the axons. We included this possible mechanism in the discussion in the revised manuscript.

It feels a bit like a missed opportunity that the experiments with the three MT regulators are not done consistently in the same system. Specifically, it would be interesting to see if tau localisation is affected by Mask in motorneurons, a context where Tau function is important (analog to the Jupiter stainings). This could be analysed either overexpressing hTau or using the endogenously tagged P{Wee-P.un}{Zhu, 2015 #572}tau304 line. Similar to the genetic interaction studies with stai and Jupiter: Does loss or gain of Tau affect mask-dependent synaptic overgrowth?

Author response: We removed the interactions between Mask and Tau from Figure 1 based on the comments from all reviewers. These data were now presented in the Supplemental material in the revised manuscript. We agree that further investigation on the interactions between Mask and Tau in the nervous system will bring insight into the mechanisms underlying their interplay. Given the fact that Tau-related neuronal dysfunction in fly neuromuscular junctions has been linked to MT-independent mechanisms such as mitochondrial defects (Chee et al., 2005 Neurobiology of Disease) and Tau-mediated interaction with synaptic vesicles (Zhou et al., 2017 Nature Communication), we decided to focus on the genetic interactions between *stai* and *mask* in the motor neurons in this manuscript, but plan to use a human Tau knock-in line (available from stock centers) together with the commercially available antibodies specific to Tau to further study the interplay between Mask and Tau in the future.

There is no experimental evidence that Mask controls MT dynamics (e.g. MT polymerisation speeds/lifetimes). Therefore, this statement should be reworded.

Author response: We will use “stability” to describe the MT-regulating function of Mask in the revised manuscript.

Out of interest: Could the authors speculate how Masks regulates MTs? By binding MTs directly, e.g. do the Mask constructs localise along MTs? Could it sequester other MTBPs?

Author response: We speculated on possible mechanisms for Mask’s action in regulating MT stability in the revised discussion session. First, there is no good evidence to support a direct control of MT properties since it is difficult to draw a conclusion on whether Mask directly binds to the MTs solely based on its cellular distribution in the cell body and in the axons. One possible mechanism is that Mask regulates MT stability through regulating Jupiter. Our data showed that the abundance of Jupiter in the axons inversely relates to Mask’s levels, but its overall level is not affected by Mask in the brain lysates. Therefore, Mask regulates the distribution of Jupiter in the axons where Jupiter likely regulates MT stability. It is unclear how Mask controls the axonal distribution of Jupiter. It might directly interact with Jupiter in the cell body and prevent it from distributing to the axons. However, using a co-immunoprecipitation or a protein tag-mediated pulldown assays between Mask and Jupiter, we could not detect stable association between the two in the larval brain lysate (data not shown). Thus, it is inconclusive whether Mask and Jupiter does not directly interact with each other, or there might be transient interaction that could affect the modification and localization of Jupiter. An alternative mechanism is that Mask regulates MT stability through interactions with other MT-associated proteins and impacts the property of the MTs in the axon, which in turn affects the binding of Jupiter to the MTs and the axonal distribution of Jupiter.

Minor points:
-Would a non-parametric test be more appropriate to be used for statistics rather than a T-test? Have the authors tested if the data are distributed normally?

© 2021. Published by The Company of Biologists under the terms of the Creative Commons Attribution License (https://creativecommons.org/licenses/by/4.0/).

Author response: We tested our data for normal distribution before we chose T-test (or one-way ANOVA) for statistical analysis.

-Figure 4A, B: stai+/- and stai-/- alone as controls are missing

Author response: New data was incorporated showing that *stai* +/- does not affect, while *stai*-/- reduces the number of boutons of the NMJs, which is consistent with previously reported characterization of *stai* mutant phenotypes (Graf et al., 2011). We added these control results to Figure 4B.

-Figure S3: Control gene is missing in the gel (A). Please provide expression values relative to control in Fig. S3B.

Author response: Figure S3 in the last submitted manuscript was now presented as Fig. S6CD. Panel A (Fig. S6C in the revised manuscript) shows the results of a RT-PCR that specifically detect Jupiter. Panel B (Fig. S6D in the revised manuscript)shows the results of quantitative RT- PCR. RPL32 was used as the reference gene in the qRT-PCR analysis, and the result was presented as relative ΔCt level above background. A better description for qRT-PCR was added to the supplemental information (Supplemental Figure Legend) in the revised manuscript.

Reviewer 3 Comments for the Author:
Overall, this is an interesting and novel story, and the data documentation appears to be of good quality. There are a few substantive issues that should be addressed before publication, but in principle, the manuscript seems appropriate for the journal.

Major issues:
1A) The use of the word "Dynamics" in the title seems inappropriate, since the authors do not show live imaging to demonstrate a change in MT s dynamic instability behavior (see 1B) -- thus, the title should be modified or new data added.

Author response: We changed the title to “Mask, the *Drosophila* Ankyrin Repeat and KH domain-containing protein, regulates microtubule stability”

1B)The authors measure MT length at the light level at a resolution where limited MT binding cannot be distinguished from single MT polymers. While transmission electron microscopy, or super-resolution (e.g. STORM) techniques, would be required to distinguish a bundled chain of MTs from a single polymer of the same length, the authors should clarify that they are measuring apparent length or total polymer fuorescence intensity in the text to avoid misleading the reader.

Author response: We have rephrased MT “length” to “apparent length”.

2) Does suppression of TauOE in Muscle reflect two additive but opposite effects on MTs that simply compensate for each other because they manifest on MT length, or a direct and specific functional relationship between Tau and Mask. Perhaps one way to distinguish might be to use generic pharmacological compounds that either stabilize or destabilize MTs in living muscle pelts (e.g. Taxol or Nocodozole) combined with perturbation in Mask and in vivo analysis of MTs. This could complement the biochemical MT extraction experiments shown in Fig 2.

Author response: We agree that further investigation on functional relationship between Mask and TauOE would bring insight into the mechanisms underlying their interplay. However, given the complexity of cellular changes trigger by Tau overexpression, we decided to make the revised manuscript simpler by focusing on the interactions between *stai* and *mask*. Our future plan is to analyze possible interactions between Mask and Tau in the nervous system (where Tau is normally expressed) by assessing how they reciprocally regulate each other’s functions. Based on the comments from all reviewers, we removed the interactions between mask and Tau from Figure 1, this data is presented in the supplemental material in the revised manuscript.

3) EB1 “comets’ are shown in Figure 3 C, however, it is not clear that the puncate hotspots here reflect polymerizing ends, or aggregates of EB1-GFP (some appear much too large to represent

© 2021. Published by The Company of Biologists under the terms of the Creative Commons Attribution License (https://creativecommons.org/licenses/by/4.0/).

MT plus ends. Moreover, there is much shaft binding of this EB1, which is only seen at levels of EB1 expression that perturb normal polymerization and dynamic instability. These data must be represented as time lapse movies to confirm that the puncta are moving at rates and directions consistent with MT polymerization. Otherwise, this panel should be removed and the text modified accordingly.

Author response: We agree with the reviewer, and the EB1::GFP data was removed.

4) One issue here is that the authors move back and forth from muscle to neuronal phenotypes, as if they assume that the Mask mechanism will function similarly in these two tissues. The Stai and Jupiter assays are performed on peripheral axon bundles, and not in muscle, but the authors do not show or clearly cite data showing that Stai and Jupiter are exclusively neuronal. To put this on context, prior analysis of multiple conserved signaling pathways and cellular processes have often shown that molecules function in quite different ways in the two cell types. This leaves the reader a bit confused when the authors turn to the question of cell type specificity in the discussion. Either a complete set of parallel assays in neurons or muscle is needed, or very careful clarification in the text.

Author response: We cited references that reported the expression profiles of Stathmin and Jupiter in the revised manuscript. Previous studies have shown that Stai is highly expressed in the nervous system, but was also detected in the early embryo and in the gonads {Ozon, 2002 #1054}{Lachkar, 2010 #1055}. Loss of function of *stai* results in reduced synaptic size of fly larval NMJs (Graf et al., 2011), as opposed to the loss of function phenotype of *mask*. Jupiter is not a neuronal specific gene, its expression was also found in the early embryos{Karpova, 2006 #993}. Our new analysis of a Jupiter GFP trap line demonstrated that Jupiter is detected in the Larval CNS (new Fig. S5).

We carefully rephrased our discussion on Mask's role in regulating MT morphology and stability. We agree that although Mask regulates MT stability in both the muscle cells and the motor neurons; there is a possibility that the downstream effectors may be different in distinct cell types.

5) Overall, the n values for NMJ sample number are very low in some genotypes (Fig 1B and 4E) – the authors should point out how many structures are being counted in each biological sample.

Author response: A better description about the structures/units chosen from each animal and used for analysis and quantification was added to the revised manuscript (Table S1). Table S1 also lists all the genotypes, corresponding N numbers and P values that were not shown in the figures or figure legends.

Minor Issues: English Grammar/Spelling

The authors should carefully proofread before submitting the revision; examples are here with correction in parentheses:

122 Our previous studies of the putative scaffolding protein Mask demonstrated that overexpressing Mask ameliorate(s) the degeneration of photoreceptors

238 Mask inhibits the abundance of the MA(MT)-associated protein Jupiter in the axons

Author response: We fixed the grammar errors and typos in the manuscript.

<u>Second decision letter</u>

MS ID#: JOCES/2021/258512

MS TITLE: Mask, the Drosophila Ankyrin Repeat and KH domain-containing protein, regulates microtubule Stability

© 2021. Published by The Company of Biologists under the terms of the Creative Commons Attribution License (https://creativecommons.org/licenses/by/4.0/).

AUTHORS: Daniel Martinez, Mingwei Zhu, Jessie J. Guidry, Niles Majeste, Hui Mao, Sarah T. Yanofsky, Xiaolin Tian, and Chunlai Wu
ARTICLE TYPE: Research Article

We have now reached a decision on the above manuscript.

To see the reviewers' reports and a copy of this decision letter, please go to: https://submit-jcs.biologists.org and click on the 'Manuscripts with Decisions' queue in the Author Area. (Corresponding author only has access to reviews.)

As you will see, the reviewers continue to raise a number of substantial criticisms that prevent me from accepting the paper at this stage. If you think that you can deal satisfactorily with the criticisms on revision, I would be pleased to see a revised manuscript.

We are aware that you may be experiencing disruption to the normal running of your lab that makes experimental revisions challenging. If it would be helpful, we encourage you to contact us to discuss your revision in greater detail. Please send us a point-by-point response indicating where you are able to address concerns raised (either experimentally or by changes to the text) and where you will not be able to do so within the normal timeframe of a revision. We will then provide further guidance. Please also note that we are happy to extend revision timeframes as necessary.

Please ensure that you clearly highlight all changes made in the revised manuscript. Please avoid using 'Tracked changes' in Word files as these are lost in PDF conversion.

I should be grateful if you would also provide a point-by-point response detailing how you have dealt with the points raised by the reviewers in the 'Response to Reviewers' box. Please attend to all of the reviewers' comments. If you do not agree with any of their criticisms or suggestions please explain clearly why this is so.

Reviewer 1

Advance summary and potential significance to field

The regulation of microtubule stability and dynamics in neurons is of great interest. The authors show that loss or gain of function of the protein Mask, a large adaptor protein, causes alterations in NMJ structure, and that mask interacts genetically with a known microtubule regulator stathmin. In muscle cells and neurons, loss of Mask shifts microtubules to a longer and more acetylated state. While the underlying mechanism is not explored, Mask could be a novel microtubule regulator.

Comments for the author

The manuscript has improved significantly, mostly because the authors removed a number of improperly done or overinterpreted experiments. I still have some reservations.
As for the Jupiter-mCh interaction, the interactions between mask and overexpressed jupiter could merely reflect effects on expression strength. Thus, the effect of mask LOF or GOF should be tested with endogenously tagged Jupiter::GFP. If this experiment verifies the result with the overexpressed Jupiter-mCherry, this would make an indirect effect via UAS-protein expression less likely (important for interpretation of a potential mechanism).

A big step towards understanding the mechanism underlying the effects that the authors are seeing would still be if we knew if Mask binds microtubules. This could be done in a differential centrifugation experiment as in figure 1, where the presence of Mask in the MT pellet could be probed in an anti-Mask Western blot. Such an experiment could also be done using UAS-overexpressed proteins in muscles.

minor comments
Figure 1, still old title even though tau data is removed
figure 2, title still says "length"

© 2021. Published by The Company of Biologists under the terms of the Creative Commons Attribution License (https://creativecommons.org/licenses/by/4.0/).

The manuscript title and several figure titles (Figure 1, 3), state that Mask "promotes" or "regulates" some aspect of microtubules. this is suggestive of a direct and modulatable effect, as in a signaling cascade. This is absolutely not known. The current descriptive data could be easily explained by a more indirect effect, e. g., through upregulation of non-specific degradation pathways like autophagy, or smetimes even through affecting GAL4 strength. More neutral expressions, such as "Mask affects microtubule stability", is more appropriate here.

Reviewer 2

Advance summary and potential significance to field

In this manuscript Martinez and colleagues describe a novel role of Drosophila Mask, a large Ankyrin repeat and KH domain containing protein in formation of MT networks in muscles and synaptic terminals in motorneurons. Using targeted expression of mask, mask-RNAi and mask mutants, the authors describe a length increase of MTs in larval muscles upon mask loss of function. They also find that loss of mask leads to an increase in presynaptic terminal growth in larval motorneurons. They furthermore describe a functional relationship between mask and two MT regulators: They observe that mask genetically interacts with Stathmin and Jupiter for the formation of presynaptic terminals. And they find that mask affects Jupiter localisation. A comprehensive structure function analysis shows that the KH domain is dispensable for mask's role in muscle MT length and synaptic terminal formation.

Overall, the authors present interesting findings and a novel role for mask that had been associated with signalling and proliferation previously. Masks therefore provides a new interesting regulator of MTs in axons and this study provides interesting findings for future mechanistic insights.

Comments for the author

Overall, the manuscript is improved. There are some concern remaining that I suggest would need to be addressed before acceptance:
- Could the authors please provide a quantification for total tubulin in Fig.2 (in addition to quantification tub levels in the pellet shown in Fig.2B) to substantiate the statement in lines 143/4 ("We found that reducing Mask levels does not alter the total ß-Tubulin levels in muscle homogenates (Fig. 2A)."). Furthermore, tubulin levels in supernatant should be quantified as well, because if overall tubulin levels are the same when mask is reduced and levels are increased in the pellet, there should be less tubulin in the supernatant.
- The re-wording from longer MTs to 'muscle cells contain more MTs that are larger in mass' is still an overinterpretation. It is not possible to distinguish in a western blot whether there are more MTs or MTs with larger mass. Please reword carefully.
Further minor points:
- Line 159: 'only pan-neuronal or ubiquitous expression, but not muscle (postsynaptic) expression, of UAS-Mask rescues the NMJ terminal overgrowth phenotypes (Fig. 3AB).: Isn't there a partial rescue, since mask10.22/Df MHC > UAS-Mask is improved in comparison to mask10.22/Df?
- Line 161: "Furthermore, neuronal knockdown of mask using mask RNAi causes similar NMJ expansions as observed in the mask genetic mutants (Fig. 3AB)." Fig reference should be Fig.4A,B
- Lines 616/7: "Figure 1, mask negatively regulates microtubule stability in larval muscle and enhances Tau-induced MT fragmentation." Tau induced fragmentation is not part of this Figure anymore.
- Lines 634/5: "Figure 3, Mask promotes normal NMJ terminal growth by regulating motor neuron microtubule stability." Regulation of stability is not shown directly those experiment. I would suggest an alternative title.
- The author response states "We changed the title to "Mask, the Drosophila Ankyrin Repeat and KH domain-containing protein, regulates microtubule stability", however, the title in the revised manuscript still states 'regulates microtubule dynamics'.

© 2021. Published by The Company of Biologists under the terms of the Creative Commons Attribution License (https://creativecommons.org/licenses/by/4.0/).

Second revision

Author response to reviewers' comments

Dear Reviewers,
On behalf of my coauthors, I would like to thank you for the opportunity to revise our manuscript JOCES/2021/258512, entitled “Mask, the Drosophila Ankyrin Repeat and KH domain-containing protein, affects microtubule stability”. We found all your comments to be helpful in further strengthening the manuscript. We have carefully considered and responded to each of your critique and suggestion, and we have incorporated the reviewers’ feedback into our revised manuscript. Below, you’ll find our responses follow below each comment in blue and are prefaced by “Author response”. Corresponding changes were also highlighted in yellow in the revised manuscript as well as the Supplemental Material. New results were presented in new Fig. 2C-E, Fig. S3 D-F, and Fig. S5 D.

Reviewer 1 Comments for the author
The manuscript has improved significantly, mostly because the authors removed a number of improperly done or overinterpreted experiments. I still have some reservations.

As for the Jupiter-mCh interaction, the interactions between mask and overexpressed jupiter could merely reflect effects on expression strength. Thus, the effect of mask LOF or GOF should be tested with endogenously tagged Jupiter::GFP. If this experiment verifies the result with the overexpressed Jupiter-mCherry, this would make an indirect effect via UAS-protein expression less likely (important for interpretation of a potential mechanism).
Author response: The effects of mask lof and gof on the endogenously tagged Jupiter::GFP were examined. The results showed that down- and up-regulation of Mask have the similar effects on the gene-trap Jupiter::GFP intensity as on the transgenic Jupiter-mCherry intensity. These results are now presented in the new Fig. S5 D & E.

A big step towards understanding the mechanism underlying the effects that the authors are seeing would still be if we knew if Mask binds microtubules. This could be done in a differential centrifugation experiment as in figure 1, where the presence of Mask in the MT pellet could be probed in an anti-Mask Western blot. Such an experiment could also be done using UAS-overexpressed proteins in muscles.

Author response: We performed microtubule co-sedimentation experiments with both larval brains and larval muscles and examined the ability of the endogenous Mask proteins to co-fractionate with taxol-induced microtubules. The experiments were repeated three times and the results consistently showed that Mask proteins are able to co-precipitate with taxol-induced MTs, either directly or indirectly. The experimental procedures were described in the revised “Material and Methods”, and the results are added in the text as well as new Fig S 3D-F.

minor comments

Figure 1, still old title even though tau data is removed
Author response: the revised title is “Mask negatively affects MT stability in larval muscle”.

figure 2, title still says "length"
Author response: the revised title is “ mask knockdown increases the sedimentation of MTs in fly larval muscles.”

The manuscript title and several figure titles (Figure 1, 3), state that Mask "promotes" or "regulates" some aspect of microtubules. this is suggestive of a direct and modulatable effect, as in a signaling cascade. This is absolutely not known. The current descriptive data could be easily explained by a more indirect effect, e. g., through upregulation of non-specific degradation pathways like autophagy, or smetimes even through affecting GAL4 strength. More neutral expressions, such as "Mask affects microtubule stability", is more appropriate here.

Author response: rewording was done and was highlighted in yellow in the revised manuscript.

© 2021. Published by The Company of Biologists under the terms of the Creative Commons Attribution License (https://creativecommons.org/licenses/by/4.0/).

Reviewer 2 Advance summary and potential significance to field
In this manuscript Martinez and colleagues describe a novel role of Drosophila Mask, a large Ankyrin repeat and KH domain containing protein in formation of MT networks in muscles and synaptic terminals in motorneurons. Using targeted expression of mask, mask-RNAi and mask mutants, the authors describe a length increase of MTs in larval muscles upon mask loss of function. They also find that loss of mask leads to an increase in presynaptic terminal growth in larval motorneurons. They furthermore describe a functional relationship between mask and two MT regulators: They observe that mask genetically interacts with Stathmin and Jupiter for the formation of presynaptic terminals. And they find that mask affects Jupiter localisation. A comprehensive structure function analysis shows that the KH domain is dispensable for mask's role in muscle MT length and synaptic terminal formation.

Overall, the authors present interesting findings and a novel role for mask that had been associated with signalling and proliferation previously. Masks therefore provides a new interesting regulator of MTs in axons and this study provides interesting findings for future mechanistic insights.

Reviewer 2 Comments for the author
Overall, the manuscript is improved. There are some concern remaining that I suggest would need to be addressed before acceptance:

- Could the authors please provide a quantification for total tubulin in Fig.2 (in addition to quantification tub levels in the pellet shown in Fig.2B) to substantiate the statement in lines 143/4 ("We found that reducing Mask levels does not alter the total β-Tubulin levels in muscle homogenates (Fig. 2A)."). Furthermore, tubulin levels in supernatant should be quantified as well, because if overall tubulin levels are the same when mask is reduced and levels are increased in the pellet, there should be less tubulin in the supernatant.

Author response: 1) The quantification of total Tubulin (normalized to Actin) is now added to Fig. 2.C. Mask knockdown in muscle does not significantly change the total Tubulin level. 2) We also quantify the Tubulin levels in the supernatant after 100 nM Taxol treatment and ultracentrifugation (Fig. 2.D), as well as what percentage of Tubulin remains in the supernatant after the 100 nM Taxol treatment and ultracentrifugation in the control and mask knockdown samples using alpha-Actin as an internal reference (Fig. 2.E). The results show that >99% of Tubulin remains in the supernatant after the 100 nM Taxol treatment and ultracentrfugation in both samples. Because of that, the Tubulin levels in the supernatant does not show significant reduction in the mask knockdown sample compared to the control.

- The re-wording from longer MTs to 'muscle cells contain more MTs that are larger in mass' is still an overinterpretation. It is not possible to distinguish in a western blot whether there are more MTs or MTs with larger mass. Please reword carefully.

Author response: ", suggesting that loss of Mask activity in muscles results in an altered MT network that comprises MT polymers with larger mass." was changed to ", suggesting that loss of Mask activity in muscles results in an altered MT network that comprises MT polymers more prone to sediment."

Further minor points:
- Line 159: 'only pan-neuronal or ubiquitous expression, but not muscle (postsynaptic) expression, of UAS-Mask rescues the NMJ terminal overgrowth phenotypes (Fig. 3AB).: Isn't there a partial rescue, since mask10.22/Df MHC > UAS-Mask is improved in comparison to mask10.22/Df?
Author response: We agree that the muscle expression of Mask does partially suppress NMJ overgrowth phenotypes. In the revised manuscript we have reworded as follows: only pan-neuronal or ubiquitous expression of UAS-Mask completely rescues the NMJ terminal overgrowth phenotypes, while muscle (postsynaptic) expression of Mask could only moderately restore the NMJ morphology (Fig. 3AB).

- Line 161: "Furthermore, neuronal knockdown of mask using mask RNAi causes similar NMJ expansions as observed in the mask genetic mutants (Fig. 3AB)." Fig reference should be Fig.4A,B
Author response: Corrected.

© 2021. Published by The Company of Biologists under the terms of the Creative Commons Attribution License (https://creativecommons.org/licenses/by/4.0/).

- Lines 616/7: “Figure 1, mask negatively regulates microtubule stability in larval muscle and enhances Tau-induced MT fragmentation.” Tau induced fragmentation is not part of this Figure anymore.
Author response: Corrected. The new title is: “mask negatively affects microtubule stability in larval muscle.”

- Lines 634/5: “Figure 3, Mask promotes normal NMJ terminal growth by regulating motor neuron microtubule stability.” Regulation of stability is not shown directly those experiment. I would suggest an alternative title.
Author response: Corrected. The new title is: Mask promotes normal NMJ terminal growth by affecting motor neuron microtubule stability.

- The author response states “We changed the title to “Mask, the Drosophila Ankyrin Repeat and KH domain-containing protein, regulates microtubule stability”, however, the title in the revised manuscript still states ‘regulates microtubule dynamics’.
Author response: Corrected. The new title is: Mask, the Drosophila Ankyrin Repeat and KH domain-containing protein, affects microtubule stability.

Third decision letter

MS ID#: JOCES/2021/258512

MS TITLE: Mask, the Drosophila Ankyrin Repeat and KH domain-containing protein, affects microtubule Stability

AUTHORS: Daniel Martinez, Mingwei Zhu, Jessie J. Guidry, Niles Majeste, Hui Mao, Sarah T. Yanofsky, Xiaolin Tian, and Chunlai Wu
ARTICLE TYPE: Research Article

I am happy to tell you that your manuscript has been accepted for publication in Journal of Cell Science, pending standard ethics checks.

© 2021. Published by The Company of Biologists under the terms of the Creative Commons Attribution License (https://creativecommons.org/licenses/by/4.0/).
